# Detrimental effects of duplicate reads and low complexity regions on RNA- and ChIP-seq data

**DOI:** 10.1186/1471-2105-16-S13-S10

**Published:** 2015-09-25

**Authors:** Mikhail G Dozmorov, Indra Adrianto, Cory B Giles, Edmund Glass, Stuart B Glenn, Courtney Montgomery, Kathy L Sivils, Lorin E Olson, Tomoaki Iwayama, Willard M Freeman, Christopher J Lessard, Jonathan D Wren

**Affiliations:** 1 Department of Biostatistics, Virginia Commonwealth University, Richmond, VA, USA; 2 Arthritis and Clinical Immunology, Oklahoma Medical Research Foundation. Oklahoma City, OK, USA; 3 Immunobiology and Cancer Research Program, Oklahoma Medical Research Foundation. Oklahoma City, OK, USA; 4 Reynolds Oklahoma Center on Aging, Donald W. Reynolds Department of Geriatric Medicine, University of Oklahoma Health Sciences Center. Oklahoma City, OK, USA; 5 Department of Physiology, University of Oklahoma Health Sciences Center. Oklahoma City, OK, USA; 6 Department of Biochemistry and Molecular Biology, University of Oklahoma Health Sciences Center. Oklahoma City, OK, USA

## Abstract

**Background:**

Adapter trimming and removal of duplicate reads are common practices in next-generation sequencing pipelines. Sequencing reads ambiguously mapped to repetitive and low complexity regions can also be problematic for accurate assessment of the biological signal, yet their impact on sequencing data has not received much attention. We investigate how trimming the adapters, removing duplicates, and filtering out reads overlapping low complexity regions influence the significance of biological signal in RNA- and ChIP-seq experiments.

**Methods:**

We assessed the effect of data processing steps on the alignment statistics and the functional enrichment analysis results of RNA- and ChIP-seq data. We compared differentially processed RNA-seq data with matching microarray data on the same patient samples to determine whether changes in pre-processing improved correlation between the two. We have developed a simple tool to remove low complexity regions, RepeatSoaker, available at https://github.com/mdozmorov/RepeatSoaker, and tested its effect on the alignment statistics and the results of the enrichment analyses.

**Results:**

Both adapter trimming and duplicate removal moderately improved the strength of biological signals in RNA-seq and ChIP-seq data. Aggressive filtering of reads overlapping with low complexity regions, as defined by RepeatMasker, further improved the strength of biological signals, and the correlation between RNA-seq and microarray gene expression data.

**Conclusions:**

Adapter trimming and duplicates removal, coupled with filtering out reads overlapping low complexity regions, is shown to increase the quality and reliability of detecting biological signals in RNA-seq and ChIP-seq data.

## Introduction

Next generation sequencing (NGS) technology is primarily based on massively parallel sequencing of millions of short reads from DNA/RNA samples, although the read length has been increasing [1,2]. The costs of NGS have rapidly dropped [3] and, consequently, there has been a relatively rapid shift from the use of microarrays to RNA-seq data to study transcription. This increased reliance on NGS necessitates examination of analysis steps that may affect the quality of the data and the interpretations drawn from it.

Although different types of NGS experiments and library preparation protocols dictate downstream processing steps, removing sequence adapters used to construct the short read library [4] as well as removing low quality bases from short reads [5] are the typical quality control steps. This is followed by removing duplicate reads, which can arise during library amplification by polymerase chain reaction [6]. The rationale behind this step is that such duplicate reads may lead to erroneous conclusions regarding the true level of biological signal, e.g., variant detection in DNA-seq data [7], gene expression in RNA-seq data [8], quantification of gene rearrangements [9], and in ChIP-seq data [10]. Two schools of thought have emerged in the field regarding how to best address this issue. The first is a widely used practice to remove all duplicate or low complexity reads from the dataset, presuming they are a source of potential bias [7,8,10-12]. The second believes that these duplicates may be true unique observations and their removal introduces bias on its own [7]. Although the effect of duplicate reads has been investigated in DNA sequencing [13], the question of how duplicates affect biological signals from gene expression in RNA-seq experiments and motif detection in ChIP-seq experiments remains open-ended.

The presence of low complexity [14,15] and repetitive elements [16] in the reference genome received less attention in how they may affect the conclusions of biological studies. Such regions complicate alignment because short reads originating from them can be mapped to multiple locations, making their interpretation challenging [17,18] or even impossible. This problem is not small, as eukaryotic genomes can be very rich in repeats; for example, some have estimated that the human genome contains ~47% repetitive regions [19]. Although recent findings from the ENCODE project suggest that the genome is pervasively transcribed [20,21], RNA molecules originating from low complexity and repetitive regions are a potential problem for analysis of RNA-seq data because they may promiscuously align throughout the genome [17]. This also causes a problem for motif detection within protein-DNA interaction regions, identified by ChIP-seq [18,22]. The use of paired-end sequencing, such as implemented by Illumina technologies, helps to control for proper mapping of reads by identifying the discordant read pairs whose mapped loci deviate from the expected orientation and insert size. The problem of multi-mapped reads has also been investigated in detection of structural variants, with the general consensus being to ignore them [23,24].

In this work, we systematically investigated the effect of adapter trimming, duplicate reads removal, and filtering out reads overlapping low complexity regions upon biological signal in RNA-seq and ChIP-seq experiments. At each processing step, we used an indirect measurement of the strength of the biological signals by performing pathway- and gene ontology enrichment analyses of genes detected from RNA-seq data, and transcription factor binding sites enrichment analyses in peaks detected from ChIP-seq data. Our rationale here is that, if a processing step leads to a more significant enrichment p-value, that processing step is likely to positively influence biological signal.

Removal of reads overlapping low complexity regions (referred hereafter as "low complexity reads") received less attention. For example, this step has been included into a set of quality control steps in PRINSEQ tool [25]. To allow isolated testing of the effect of low complexity reads removal, we introduce a simple post-alignment filtering tool, RepeatSoaker, that filters out reads overlapping with a user provided template file which contains genomic coordinates of low complexity regions. Designed to be aligner-independent, RepeatSoaker processes the aligned data in BAM format, removes low complexity reads, and outputs a cleaned BAM file and filtering statistics. RepeatSoaker is a straightforward method to remove alignment artifacts from NGS data, designed to eliminate potential false positive reads in quantifying transcript expression. Extendable to any other sequencing technology where low complexity reads may introduce bias, such as ChIP-seq, we envision RepeatSoaker could become a standard step helping to better structure reproducible NGS pipelines.

We applied a combination of adapter trimming, duplicate removal, and filtering out low complexity reads with RepeatSoaker to RNA-seq and ChIP-seq experiments, and investigated how each step affects the results of downstream enrichment analysis. Our results show that adapter trimming increases the significance of gene ontology and pathway enrichment analyses in RNA-seq data, and strengthens motif detection in ChIP-seq data. The duplicate removal step, despite decreasing the number of reads, further helps to increase the significance of biological signals, especially when coupled with adapter trimming. Filtering out low complexity reads with RepeatSoaker has minor effect on the total number of reads, yet this step had a positive effect on the detection of biological signals. Our study suggests that adapter trimming and duplicates removal are important steps in detecting stronger biological signals within RNA-seq and ChIP-seq data, and optional filtering out of reads overlapping low complexity regions will further increase the quality of conclusions.

## Methods

### Data source

The effect of adapter trimming, duplicate removal and filtering out reads overlapping low complexity region was investigated in 2 types of data: RNA-seq and ChIP-seq.

Human RNA-seq data were obtained from 60 Sjogren's syndrome patients and 30 healthy controls (unpublished data). In short, peripheral blood was collected in PaxGene tubes (BD Diagnostics; Franklin Lakes, NJ) and RNA was isolated using standard protocols (Qiagen, Inc., Valencia, CA). Globin transcripts were removed using GlobinClear (Life Technologies; Grand Island, NY) and samples were prepared for sequencing using the NuGen ENCORE complete kit (San Carlos, CA). The paired-end 100 bp-long sequencing was performed using the Illumina HiSeq 2000 employing standard procedures. For the 54 samples used in RNA-seq experiment, matching gene expression profiling was performed using Illumina HumanWG-6 v3.0 BeadChips. Pearson's correlation coefficient was used to test the agreement between human RNA expression estimates measured by RNA-Seq (log2 FPKM counts) and microarrays (log2 intensities) in the cohort of patients diagnosed with Sjogren's syndrome.

Mouse RNA-seq data were obtained from 3 wild type mice and 3 mice expressing the D842V mutant form of PDGFRα (platelet-derived growth factor receptor alpha), which was previously described [26]. Cell suspensions from neonatal dermis were prepared from 3 day old mouse skin after separating dermis/epidermis and digesting the dermis with 0.35% collagenase type 1 for 60 minutes. Subsequently, Nestin-GFP+ singlets were sorted with a MoFlo XDP cell sorter (Beckman Coulter). RNA was isolated from 2-3 million cells using RNAeasy kit (Qiagen). cDNA libraries were prepared with NEBNext Ultra Directional RNA Library Prep Kit for Illumina (New England Biolabs) according to the manufacturer's protocol. In short, mRNA was isolated from 1µg purified total RNA with oligo dT beads, and fragmented. Then, first and second strand cDNA were synthesized, followed by purification using Agencourt AMPure XP beads (Beckman Coulter). The second strand cDNAs were end-repaired, A-tailed, and adaptor-ligated. Size-selected DNA with Agencourt AMPure XP beads was enriched by 13-cycle PCR each with index primers, and again purified using the beads. The each indexed library was analyzed by Agilent 2200 TapeStation system (Agilent), and RNA integrity number equivalent (RINe) were ranged from 9.2 to 9.6. Then libraries were quantified with Qubit 2.0 Fluorometer (Thermo Fisher Scientific), and pooled for sequencing.

ChIP-seq data were obtained from 10 systemic lupus erythematosus patients and 10 healthy controls of European descent (unpublished data). Briefly, all nucleated cells were isolated from human blood using PolyPrep (Sigma, Deisenhofen, Germany) density gradient medium. Proteins were cross-linked to the DNA using formaldehyde and protein-DNA complexes were immunoprecipitated using a polyclonal rabbit anti-PU.1 (Spi-1) antibody (sc-352, Santa Cruz Biotechnology, Santa Cruz, California, USA). Individual sequencing libraries were prepared for each individual using the Illumina ChIP-Seq DNA Sample Prep Kit (Illumina, San Diego, California, USA). Sequencing was done using the Illumina HiSeq 2000 platform with 5 samples per lane. The case-control samples were sequenced on the same lane, e.g., 3 cases+2 controls in one lane.

### Data processing

Quality of raw sequence data was assessed using FASTQC. Adapter trimming was performed using Trimmomatic v0.30 program [27]. The reads were cropped to the length of 70 bp, the adapters were trimmed, and bases having quality below 20 on Phred 33 scale were also cut. The reads with length less than 10 bases were discarded. Only the paired reads were used for subsequent analyses. Duplicates removal was performed using PICARD MarkDuplicates tool. The summary statistics were obtained using SAMTOOLS FLAGSTAT command [11].

To remove reads overlapping any of the regions identified by RepeatMasker [28,29], we implemented RepeatSoaker (https://github.com/mdozmorov/RepeatSoaker). RepeatSoaker utilizes a user-provided list of genomic coordinates of low complexity regions in BED format [30]. Note that the coordinates should correspond to the organism and genome assembly of the original BAM files. We provide a Makefile that automates generation of BED files containing genomic coordinates of all regions identified by RepeatMasker for GRCh37/hg19 and NCBI37/mm9 genomes.

RepeatSoaker provides flexibility to set a threshold for deciding whether a read should be kept or filtered due to its overlap with a low complexity region. A user can set the percent overlap threshold, e.g., 75%, 50%, 25%. A read overlapping with a low complexity region more than the threshold, e.g., more than 75%, is filtered. We tested the effect of removing reads overlapping with low complexity regions more than 75%, 50%, 25%, and 0%. A 0% threshold indicates that a read is filtered if it is immediately proximal to a repeat region.

For the human and mouse RNA-seq data, raw FASTQ files were aligned to the human and mouse genomes (hg19/mm9, respectively) using TOPHAT [31]. The read counts per gene or transcript were generated using HTSEQ-COUNT. Differentially expressed (DE) transcripts were determined using DESeq R package with a false discovery rate (FDR) q-value of <0.05 and a fold change of >2 or <0.5. All data manipulations were performed in the R/Bioconductor environment [32].

For the human ChIP-seq data, raw FASTQ files were aligned to the human hg19 genome using bowtie2 [33]. The PU.1 binding peaks were called using MACS2 [34], and the consensus motifs were detected using MEME-ChIP suite [35].

## Results

### Systematic testing of data preprocessing steps

**Figure 1:**
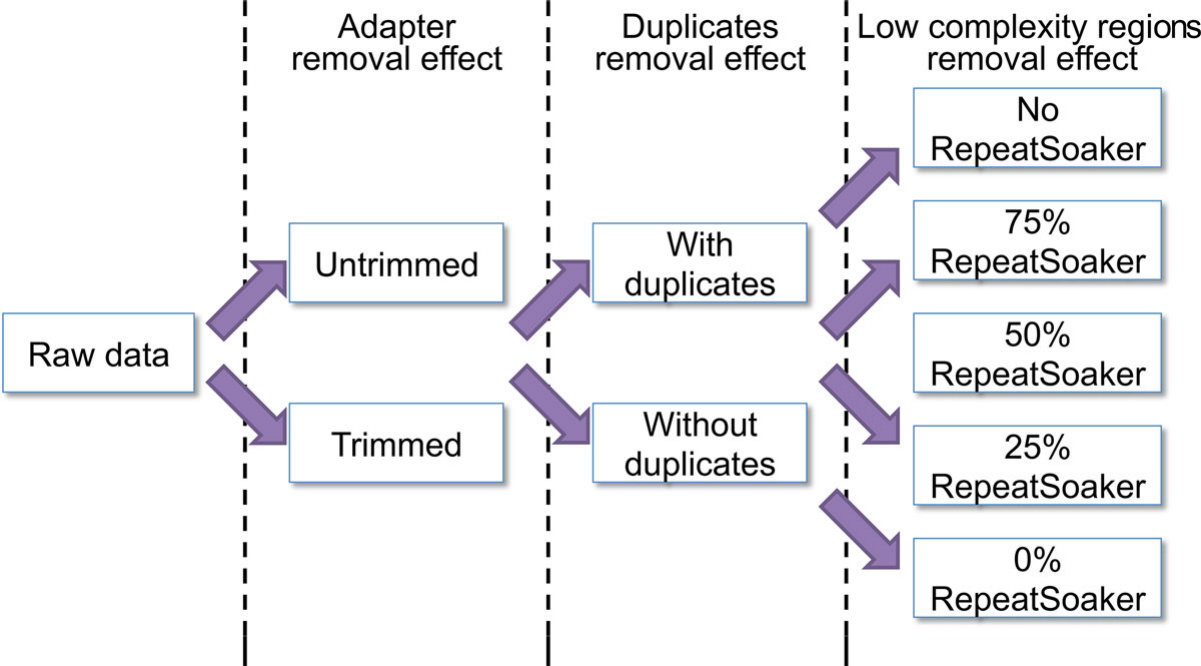
**RepeatSoaker comparisons**. Overview of the various permutations of the three data processing steps compared.

To elucidate the effects of adapter removal, elimination of duplicates, and filtering of low complexity regions, we performed systematic testing of sequencing data with and without applying these three preprocessing steps (Figure 1). At each step, we compared the alignment statistics, the number of differentially expressed genes (RNA-seq) or identified transcription factor binding peaks (ChIP-seq), and the results of Gene Ontology, KEGG and Reactome pathway enrichment analyses (RNA-seq) and motif enrichment analyses (ChIP-seq). We also compared combinations of data preprocessing steps, e.g., how duplicate removal affected trimmed and untrimmed data. At each comparison, we evaluated how the data preprocessing steps affected biological signals as judged by the functional enrichment analysis.

### Adapter removal increases the data quality and the strength of biological signals

Before investigating the effects of low complexity region filtering, we assessed how adapter- and duplicate removal affected quality of alignment of the sequencing data. Adapter trimming increased the number of total reads in RNA-seq data, due to the fact that Trimmomatic had cut low quality bases from the middle portion of some of the reads, thus splitting some of them into multiple shorter reads, which still survived the minimum length (10 bp) threshold (Table 1 Additional Files 1 and 2). Such cuts resulted in more reads with mates mapped to a different chromosome (referred hereafter as "mismapped reads"). Adapter trimming of ChIP-seq data slightly decreased the total number of reads, as compared with unprocessed data (Additional File 3). However, the percentage of properly paired reads increased while the percent of singletons and mismapped reads decreased (Table 2). In summary, the adapter-trimming step altered the properties of sequencing data depending on the quality of the unprocessed data.

**Table 1:** RNA-seq alignment statistics for different combinations of the sequencing data processing steps

Trim	Dup	RS	Total reads	properly paired (%)	singletons (%)	with mate mapped to a different chr (%)	Number of DEGs	KEGG: ECM-receptor interaction	GO: multicellular organismal process	Reactome: Transmembrane transport of small molecules	R^2^
-	-	-	41,505,942	68.98+-3.71	16.03+-3.20	4.01+-3.48	2189	1.86E-07	8.86E-16	6.38E-13	0.6687

+	-	-	51,984,539	60.10+-3.68	10.98+-2.44	17.92+-3.89	2139	3.29E-08	2.86E-13	9.85E-11	0.6614

-	+	-	15,429,501	61.72+-5.45	12.49+-2.37	10.22+-4.68	2487	1.34E-07	2.50E-22	1.85E-11	0.6672

+	+	-	25,738,167	43.14+-5.77	7.51+-1.33	36.18+-5.70	2391	1.97E-07	4.93E-17	3.54E-09	0.6575

-	-	75	28,283,010	70.55+-3.17	16.74+-3.85	0.69+-0.09	2100	7.62E-08	8.85E-17	5.71E-12	0.6708

-	-	50	26,450,592	70.05+-3.24	17.22+-3.95	0.63+-0.08	2068	7.18E-08	1.31E-16	6.85E-14	0.6712

-	-	25	24,703,408	69.63+-3.31	17.66+-4.05	0.62+-0.08	2021	8.92E-09	6.33E-19	2.94E-14	0.6705

-	-	0	21,413,178	69.39+-3.47	18.20+-4.26	0.61+-0.09	2087	1.02E-08	3.13E-19	3.98E-15	0.6643

+	-	75	32,589,028	64.70+-3.88	12.29+-2.88	10.46+-1.82	2116	4.88E-07	1.12E-13	2.89E-12	0.6637

+	-	50	30,174,345	64.93+-3.92	12.70+-2.97	9.71+-1.82	2066	3.49E-07	2.96E-14	2.44E-12	0.6642

+	-	25	28,231,486	64.55+-4.00	13.04+-3.03	9.76+-1.87	2004	3.15E-07	5.05E-16	1.13E-13	0.6636

+	-	0	24,546,936	63.85+-4.25	13.42+-3.12	10.26+-2.06	2028	2.65E-07	3.42E-16	3.55E-14	0.6583

-	+	75	9,681,047	68.59+-4.15	12.22+-2.96	1.45+-0.25	2302	1.21E-07	4.18E-23	5.71E-12	0.6695

-	+	50	8,987,150	68.34+-4.18	12.53+-3.06	1.221+-0.20	2256	1.48E-07	4.80E-22	4.07E-14	0.6700

-	+	25	8,346,861	68.09+-4.22	12.83+-3.16	1.21+-0.19	2245	1.48E-07	2.70E-21	1.44E-14	0.6694

-	+	0	7,151,500	68.13+-4.26	12.99+-3.12	1.19+-0.20	2326	1.69E-07	2.81E-24	8.28E-16	0.6628

+	+	75	14,251,402	52.88+-6.50	7.54+-1.45	23.48+-5.56	2210	1.18E-06	4.34E-20	3.95E-09	0.6598

+	+	50	12,985,873	53.93+-6.45	7.65+-1.48	22.01+-5.48	2180	7.69E-07	5.94E-19	3.02E-11	0.6604

+	+	25	12,125,100	53.69+-6.50	7.81+-1.51	22.11+-5.58	2124	4.40E-06	2.98E-19	6.28E-13	0.6599

+	+	0	10,416,970	52.34+-6.98	7.74+-1.30	23.54+-6.17	2176	4.22E-06	3.54E-18	3.00E-13	0.6539

"Total reads" - average number of reads; "paired (%)" - average percent of paired reads; "singletons (%)" - average percent of single end reads; "with mate mapped to a different chr (%)" - average percent of inter-chromosome mapped reads. "Number of DEGs" - number of differentially expressed genes. To allow direct comparisons of p-values among the processing steps, the "ECM-receptor interaction" KEGG pathway, the "multicellular organismal process" GO, and the "Transmembrane transport of small molecules" Reactome pathway were selected as the most representative and most enriched functional categories in each processing step, with the full enrichment analyses results shown in Additional Files 4 and 5. "+/-" indicate whether the step (Trim - adapter trimming, Dup - duplicate removal, RS - filtering out low complexity regions with RepeatSoaker) was applied/not applied, respectively. The number in the RepeatSoaker column reflects the threshold of removing reads overlapping with low complexity regions, i.e., 75% indicates that reads overlapping 75% or more with a low complexity region were removed.

**Table 2:** ChIP-seq alignment statistics for different combinations of sequencing data processing steps

Trim	Dup	RS	Total reads	properly paired (%)	singletons (%)	with mate mapped to a different chr (%)	SPI1 E-value	Number of motifs
-	-	-	70,929,429	96.81+-1.57	0.40+-0.17	0.80+-0.34	8.2e-9446	44

+	-	-	70,155,620	97.49+-1.44	0.15+-0.03	0.39+-0.13	7.6e-10075	65

-	+	-	40,472,954	95.60+-2.19	0.44+-0.24	1.53+-1.06	1.5e-10726	26

+	+	-	40,500,416	96.83+-1.82	0.16+-0.04	0.65+-0.30	4.0e-11010	25

-	-	75	68,856,152	96.81+-1.57	0.39+-0.16	0.79+-0.34	2.0e-9425	43

-	-	50	68,578,937	96.81+-1.57	0.39+-0.16	0.79+-0.34	2.0e-9425	42

-	-	25	68,405,768	96.81+-1.57	0.39+-0.16	0.79+-0.34	2.0e-9425	43

-	-	0	68,279,169	96.81+-1.57	0.39+-0.16	0.79+-0.34	2.0e-9425	42

+	-	75	68,004,984	97.50+-1.45	0.15+-0.03	0.38+-0.13	2.8e-9899	64

+	-	50	67,805,679	97.50+-1.45	0.15+-0.03	0.38+-0.13	2.8e-9899	64

+	-	25	67,679,663	97.50+-1.45	0.15+-0.03	0.38+-0.13	2.8e-9899	67

+	-	0	67,587,630	97.50+-1.45	0.15+-0.03	0.38+-0.13	2.8e-9899	62

-	+	75	39,242,893	95.61+-2.19	0.43+-0.24	1.51+-1.05	7.6e-10575	26

-	+	50	39,080,973	95.61+-2.19	0.43+-0.24	1.51+-1.05	7.6e-10575	26

-	+	25	38,981,300	95.61+-2.19	0.43+-0.24	1.51+-1.05	7.6e-10575	26

-	+	0	38,908,929	95.61+-2.19	0.43+-0.24	1.51+-1.05	7.6e-10575	26

+	+	75	39,242,893	95.61+-2.19	0.43+-0.24	1.51+-1.05	4.7e-10731	27

+	+	50	39,080,973	95.61+-2.19	0.43+-0.24	1.51+-1.05	4.7e-10731	27

+	+	25	38,981,300	95.61+-2.19	0.43+-0.24	1.51+-1.05	4.7e-10731	27

+	+	0	38,908,929	95.61+-2.19	0.43+-0.24	1.51+-1.05	4.7e-10731	30

"+/-" indicate the step (Trim - adapter trimming, Dup - duplicate removal, RS - filtering out low complexity regions with RepeatSoaker) was applied/not applied, respectively. The RepeatSoaker % reflects the threshold of removing reads overlapping with low complexity regions, i.e., 75% indicates that reads overlapping with low complexity regions 75% or more are removed. SPI1 E-value is an equivalent of a p-value for the detection of PU.1 motif.

To identify how the adapter-trimming step affects biological signals in RNA-seq data, we evaluated the total number of differentially expressed genes before and after trimming, and their functional enrichment results. The total number of differentially expressed genes remained virtually unchanged, as well as the order of enrichment p-values for KEGG, GO, and Reactome pathway enrichment results, and Pearson's correlation coefficient with the microarray gene expression data (Table 1 Additional Files 4 and 5). In the case of ChIP-seq experiments, we evaluated the number and the significance of detected motifs within ChIP-seq binding peaks. As we have evaluated genome-wide binding of the PU.1 protein, the most significant motif enriched in the detected peaks was SPI1 (aka PU.1, Additional File 6). The total number and the significance of the detection of this motif increased following the adapter trimming step. These results support the notion that adapter trimming alone increases the strength of biological signals within it [5].

### Removing duplicates negatively affects alignment statistics but strongly improves the detection of biologically relevant signal

Removing duplicates had the greatest effect on the alignment statistics, decreasing the total number of reads by ~40% in RNA-seq and ChIP-seq data, as compared with the unprocessed data (Additional Files 1, 2, 3). This step also decreased the percentage of singletons (Tables 1 and 2). Yet, this step had a slight negative effect on the percentage of properly paired reads at the expense of increased percentage of mismapped reads. Overall, the effect of duplicates removal on the alignment statistics was detrimental.

Despite the lower number of short reads remaining after duplicates removal, the number of differentially expressed genes increased (Table 1). Although the lists of differentially expressed genes before and after duplicate removal did not show complete overlap (Additional Files 4 and 5), the order and the significance of the enriched KEGG, GOs and Reactome pathways remained similar indicating that the biological signal was retained and became more significant, as the enrichment p-values were also increased (Table 1). We did not observe any increase in correlation of RNA-seq and microarray gene expression data.

In contrast to a larger number of differentially expressed genes when removing duplicates from the data, the number of detected motifs in ChIP-seq data decreased. However, the significance of the detected PU.1 motif increased (Table 2 Additional File 6). In summary, these results suggest that removal of duplicates, although decreasing the total number of reads, increases the significance of biological signals in both RNA-seq and ChIP-seq data.

### Trimming adapters coupled with duplicates removal synergistically improves the quality of sequencing data

Adapter trimming coupled with duplicates removal led to an increase in the percentage of mismapped reads in RNA-seq data (Table 1). Consequently, the percentage of properly paired reads has decreased. However, the number of differentially expressed genes was larger than in the unprocessed data and their enrichment analyses results, except for the adapter-trimming step, were comparably significant.

Applied to ChIP-seq data, trimming the adapters coupled with removing duplicates improved overlap statistics, making them better than the unprocessed data (Table 2). Notably, despite lower total number of detected motifs, the detection of the PU.1 motif was the most significant (Additional File 6). Overall, our results suggest that both adapter trimming and duplicates removal steps help to emphasize biological signal in RNA-seq and ChIP-seq data.

### Removing low complexity regions improves detection of true biological signal

We used different stringency thresholds for filtering reads overlapping low complexity regions. A threshold was defined by the percent of overlap of a read with low complexity regions. For example, 75% threshold indicates that a read will be filtered only if it overlaps with a low complexity region at least 75% of the read's total length. A special case of 0% threshold indicates that a read can be located side-by-side with a low complexity region to be considered for removal. Thus, lowering percent overlap threshold corresponds to a more stringent threshold for filtering out of reads overlapping low complexity regions, with 0% threshold indicating the most aggressive filtering.

Applying RepeatSoaker to RNA-seq and ChIP-seq data decreased the number of reads by ~3%, independently of the threshold used. However, the alignment statistics of RNA-seq and ChIP-seq data improved in all conditions, when compared with the corresponding data preprocessing steps. For example, comparing the alignment statistics for unprocessed and processed (filtered) data shows not only increase in the percentage of properly paired reads, but also in decrease in mismapped reads (Additional Files 1, 2, 3). Increasing the stringency of filtering of the reads overlapping low complexity regions further increased the alignment statistics. This suggests that simply removing reads overlapping low complexity regions is an essential step in improving the quality of RNA- and ChIP-seq data.

Reflecting the improvement in the alignment statistics, the significance of the GO/KEGG/Reactome enrichments (RNA-seq) and the PU.1 motif enrichments (ChIP-seq) have increased upon RepeatSoaking the data (Table 2), while the order of the enrichments remained virtually unchanged. Moreover, more stringent removal of reads overlapping low complexity regions further increased the significance of the functional enrichments. Correlation of gene expression from RNA-seq and microarray data was also increased. This observation suggests that aggressive removal of reads overlapping low complexity regions (RepeatSoaker threshold 0%) aids in emphasizing true biological signal within the data.

### Filtering out low complexity reads affects low expressed metabolism-related genes

**Figure 2:**
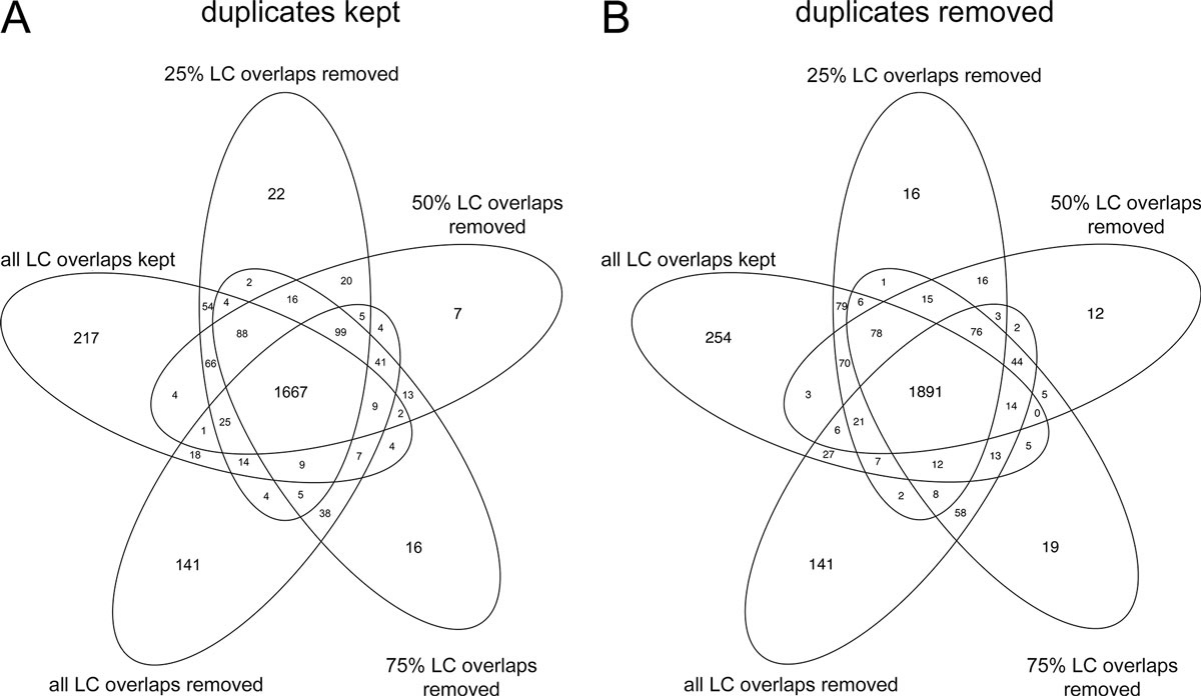
**Differential expression detection**. Number of differentially expressed genes detected after removing reads overlapping low complexity (LC) regions. Conditions for differential expression analysis: "all LC overlaps kept/removed" - reads touching/overlapping LC regions are either kept or removed, respectively; "25%/50%/75% LC overlaps removed" - reads overlapping LC regions at least 25%/50%/75%, respectively, are removed before differential expression analysis.

**Figure 3:**
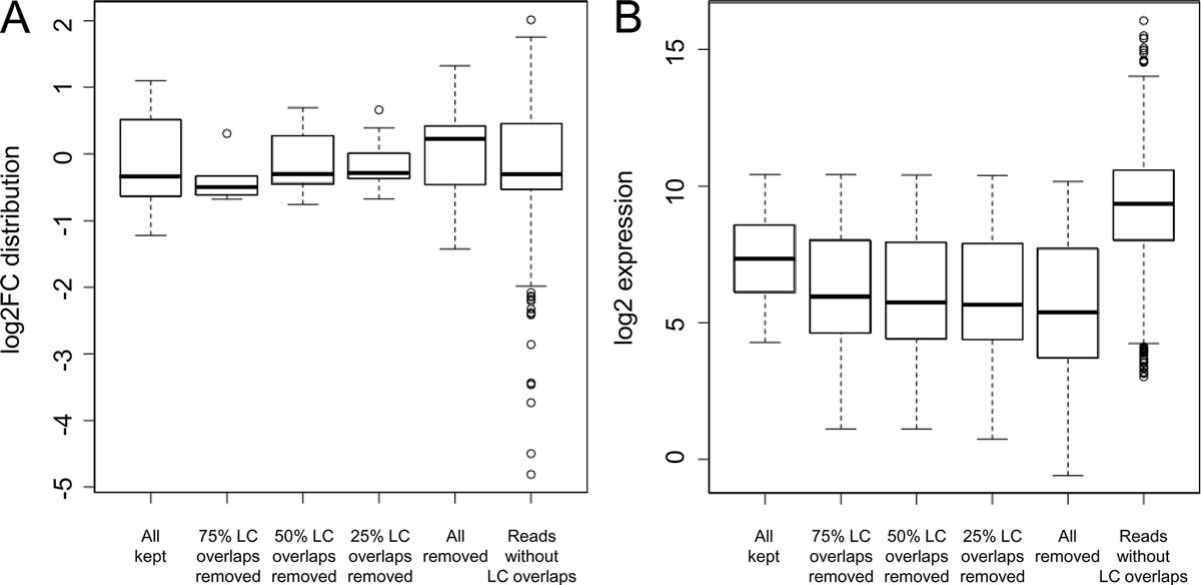
**Gene comparison of distribution and expression levels**. Comparison of the log2 fold change (A) and expression (B) distributions among genes at different thresholds for removing reads overlapping low complexity (LC) regions. "All kept"/"Reads without LC overlaps" - metrics of all differentially expressed genes detected using all/none reads overlapping LC regions; "75%/50%/25% LC overlaps removed" - metrics of genes that became non-differentially expressed after removing reads overlapping LC regions at least 75%/50%/25%, respectively.

We investigated the effect of RepeatSoaker on the number of differentially expressed (DE) genes in RNA-seq data with and without duplicates (Figure 2). The total number of DE genes decreased with more stringent removal of reads with RepeatSoaker. Each condition, from no RepeatSoaker to the most stringent RepeatSoaker threshold 0%, has a unique set of genes not detected as differentially expressed in other conditions ("leaves" of the Venn diagram, Figure 2). These genes showed comparable fold change distributions as the "core" gene set detected as DE in all conditions (Figure 3A). However, the expression level of those genes was lower (Figure 3B), making them susceptible to lose their DE status upon removal of reads with RepeatSoaker. We further investigated the biological significance of those genes. These were predominantly predicted (as opposed to known) genes and metabolism-related genes, as can be seen from their enrichment analysis results (Additional File 7). This observation suggests that RepeatSoaker removes biological "noise" from the data while increasing the signal that reflects the underlying biology of the experiment.

### Removing duplicates decreases overall expression level but retains fold changes

**Figure 4:**
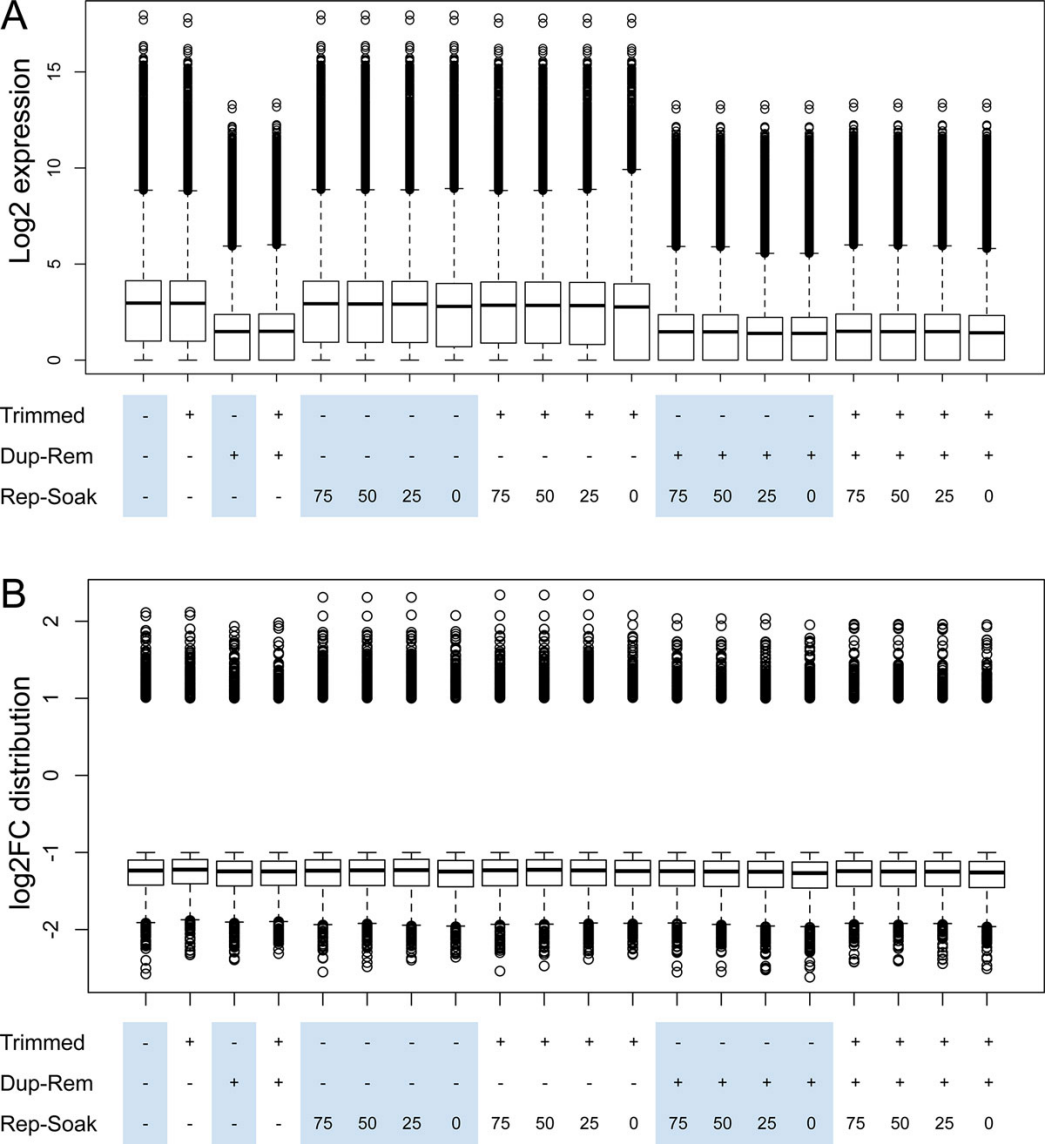
**Gene comparison of distribution and expression levels**. Effect of data processing on expression (A) and fold change (B) distribution of differentially expressed genes.

Lastly, we investigated the effect of data preprocessing steps upon the expression and fold change levels in RNA-seq data. Removing duplicates had decreased the overall level of expression (Figure 4A), as can be expected from losing ~40% of reads. The effect of other processing steps on gene expression level was negligible, which is reflected in the virtually unchanged Pearson's correlation coefficient of RNA-seq and microarray gene expression data.

To compare the effect of preprocessing steps on fold changes, we compared the distribution of fold changes at each step (Figure 4B), and investigated gene-by-gene fold change differences. As pre-processing steps were applied to both healthy and diseased groups of samples, thus uniformly changing read counts of the differentially expressed genes in both groups, the fold changes remained stable (Figure 4B). Expectedly, removing duplicates, with or without adapter trimming, decreased the maximum, but not overall, fold change, as would be expected from removing ~40% of reads. Similarly, filtering out reads overlapping and touching low complexity regions (RepeatSoaker threshold 0%) also slightly decreased the maximum fold change. Overall, our results suggest that data preprocessing steps, although affecting overall gene expression level, retain biological signal within the data, as reflected by relatively unchanged fold changes.

## Discussion

Our findings suggest some general guidelines for RNA-seq and ChIP-seq data processing. We show that each of the three steps, adapter trimming, duplicate removal, and filtering out reads overlapping low complexity regions, increases the strength of biological signals within the data, as shown by the more significant functional enrichment p-values. Our results emphasize the need of removing reads overlapping low complexity regions in order to improve biological signals in RNA-seq and ChIP-seq data. Overall, our analysis suggests that all three steps should be an integral part of NGS data processing pipelines in order to obtain better insights into the biology.

Although it is a general consensus that trimming the adapters improves the quality of sequencing data [5], our experience with applying adapter trimming to RNA-seq and ChIP-seq data has been mixed. Trimming the adapters did not significantly improve the significance of biological signals of RNA-seq data (Table 1), in contrast with what we observed for ChIP-seq data. This may be attributed to the use of different aligners, TopHat and Bowtie2, used for RNA-seq and ChIP-seq data, respectively. The former, TopHat, has been designed to deal with unmapped portions of the reads, such as adapters [31], and therefore the data processed with it may not be notably improved by the adapter trimming step. Our results warrant the testing of other adapter trimming tools, each reported to have different effect on data quality [5].

Our motivation to investigate the effect of reads overlapping low complexity regions came from our and others [22] empirical observations that such reads have multiple alignments within the reference genome and tend to pile up within low complexity regions, such as centromeres and telomeres. We hypothesized that such pileups may negatively affect the detection of true gene expression level when summarizing read counts into FPKM measurements. Furthermore, such pileups were picked up as the strongest peaks in ChIP-seq experiments, ultimately affecting motif enrichment analysis. To this end we have developed the RepeatSoaker tool that filters out reads overlapping low complexity regions. In addition to using low complexity regions defined by the RepeatMasker program [28], a user has an option to provide his/her own list of genomic coordinates of any other regions, such as the Duke excluded regions or the DAC blacklisted regions, defined by the ENCODE project and obtainable from the UCSC genome database [36], or the high-depth coverage regions defined by Pickrell et.al. by scanning the 1000 Genomes data [22]. This ability of RepeatSoaker to use any list of genomic regions as a "mask" further empowers a user to ignore reads not only in the low complexity regions, but in other uninteresting regions, such as ribosomal genes in RNA-seq data, or even completely mask out non-exome portions of the genome.

One limitation of our study is that we cannot judge the biological significance of our findings other than by indirect assessment of the number of differentially expressed genes (RNA-seq), the total number of motifs (ChIP-seq), and the results of the enrichment analyses. Our hypothesis here was that, if a processing step improves the quality of the data, it should improve the significance of enriched gene ontologies, KEGG and Reactome pathways (for RNA-seq data) and detected motifs (for ChIP-seq data). Although we observed improvements in the enrichment analyses results after each data preprocessing steps, future studies warrant investigation of expression level of genes removed by data preprocessing steps, and by RepeatSoaker, by other techniques, such as polymerase chain reaction, or by direct comparison with gene expression changes measured by microarray technology.

## Conclusions

In conclusion, we recommend adapter trimming, duplicates removal, and filtering out reads overlapping low complexity regions as data preprocessing steps of RNA-seq and ChIP-seq data. Our comprehensive comparison suggests that these data preprocessing steps will help to emphasize true biological signals in sequencing studies.

## Competing interests

The authors declare that they have no competing interests.

## Authors' contributions

Conceived and designed the experiments: JDW, MGD. Provided the data: CM, KLS, LEO, TI, WMF, CJL. Analyzed the data: MGD, IA, CBG, SBG. Wrote the first draft of the manuscript: MGD. Made critical revisions and approved final version: JDW, IA, CL, CBG, EG. All authors reviewed and approved the final manuscript.

## Supplementary Material

Additional File 1 **Alignment statistics for the non-trimmed RNA-seq data in each data preprocessing step**. Each worksheet contains step-specific alignment statistics. "no_remDup"/"remDup" in the worksheet name indicate whether the data has duplicates kept/removed, respectively. "All"/"0.75" etc., indicate whether the reads overlapping low complexity regions were kept/removed at the specified threshold, respectively Click here for file

Additional File 2 **Alignment statistics for the trimmed RNA-seq data in each data preprocessing step**. Each worksheet contains step-specific alignment statistics. "no_remDup"/"remDup" in the worksheet name indicate whether the data has duplicates kept/removed, respectively. "All"/"0.75" etc., indicate whether the reads overlapping low complexity regions were kept/removed at the specified threshold, respectively. Click here for file

Additional File 3 **Alignment statistics for the ChIP-seq data in each data preprocessing step**. Click here for file

Additional File 4 **Differentially expressed genes in the non-trimmed RNA-seq data, and the results of KEGG/GO/Reactome pathway enrichment analyses identified in each data preprocessing step**. Each worksheet contains step-specific alignment statistics, abbreviations are the same as in the Additional File 1. Click here for file

Additional File 5 Differentially expressed genes in the trimmed RNA-seq data, and the results of KEGG/GO/Reactome pathway enrichment analyses identified in each data preprocessing step. Each worksheet contains step-specific alignment statistics, abbreviations are the same as in the Additional File 2. Click here for file

Additional File 6 **Motifs and their detection E-values identified in the ChIP-seq data in each data preprocessing step**. Click here for file

Additional File 7 **Investigation of the biology of genes lost after filtering out low complexity reads with RepeatSoaker**. Click here for file

## References

[B1] Huddleston J Ranade S Malig M Antonacci F Chaisson M Hon L Reconstructing complex regions of genomes using long-read sequencing technology Genome Res 2014 24 4 688 696 10.1101/gr.168450.113 24418700 PMC3975067

[B2] Howorka S Cheley S Bayley H Sequence-specific detection of individual DNA strands using engineered nanopores Nat Biotechnol 2001 19 7 636 639 10.1038/90236 11433274

[B3] DNA sequencing costs http://www.genome.gov/sequencingcosts/

[B4] Walsh P Lu X Carroll J An Analysis of Next Generation Sequence Clipping Tools Collaborative European Research Conference CERC 2013 2013

[B5] Del Fabbro C Scalabrin S Morgante M Giorgi FM An extensive evaluation of read trimming effects on Illumina NGS data analysis PLoS One 2013 8 12 e85024 10.1371/journal.pone.0085024 24376861 PMC3871669

[B6] Turner S Armstrong LL Bradford Y Carlson CS Crawford DC Crenshaw AT Jonathan L Haines Quality control procedures for genome-wide association studies Current protocols in human genetics / editorial board 2011 Chapter 1:Unit11.19 10.1002/0471142905.hg0119s68 PMC3066182 21234875

[B7] DePristo MA Banks E Poplin R Garimella KV Maguire JR Hartl C A framework for variation discovery and genotyping using next-generation DNA sequencing data Nat Genet 2011 43 5 491 498 10.1038/ng.806 21478889 PMC3083463

[B8] How PCR Duplicates Arise in Next-Generation Sequencing http://www.cureffi.org/2012/12/11/how-pcr-duplicates-arise-in-next-generation-sequencing/

[B9] Abel HJ Al-Kateb H Cottrell CE Bredemeyer AJ Pritchard CC Grossmann AH Detection of Gene Rearrangements in Targeted Clinical Next-Generation Sequencing J Mol Diagn 2014 16 4 405 417 10.1016/j.jmoldx.2014.03.006 24813172 PMC4078366

[B10] Chen Y Negre N Li Q Mieczkowska JO Slattery M Liu T Systematic evaluation of factors influencing ChIP-seq fidelity Nat Methods 2012 9 6 609 614 10.1038/nmeth.1985 22522655 PMC3477507

[B11] Li H Handsaker B Wysoker A Fennell T Ruan J Homer N The Sequence Alignment/Map format and SAMtools Bioinformatics 2009 25 16 2078 2079 10.1093/bioinformatics/btp352 19505943 PMC2723002

[B12] Furey TS ChIP-seq and beyond: new and improved methodologies to detect and characterize protein-DNA interactions Nat Rev Genet 2012 13 12 840 852 10.1038/nrg3306 23090257 PMC3591838

[B13] Zhou W Chen T Zhao H Eterovic AK Meric-Bernstam F Mills GB Chen K Bias from removing read duplication in ultra-deep sequencing experiments Bioinformatics 2014 10.1093/bioinformatics/btt771 PMC3982159 24389657

[B14] Majewski J Ott J Distribution and characterization of regulatory elements in the human genome Genome Res 2002 12 12 1827 1836 10.1101/gr.606402 12466286 PMC187578

[B15] DePristo MA Zilversmit MM Hartl DL On the abundance, amino acid composition, and evolutionary dynamics of low-complexity regions in proteins Gene 2006 378 19 30 16806741 10.1016/j.gene.2006.03.023

[B16] Kapitonov VV Jurka J A universal classification of eukaryotic transposable elements implemented in Repbase Nat Rev Genet 2008 9 5 411 412 10.1038/nrg2165-c1 18421312

[B17] Li B Ruotti V Stewart RM Thomson JA Dewey CN RNA-Seq gene expression estimation with read mapping uncertainty Bioinformatics 2010 26 4 493 500 10.1093/bioinformatics/btp692 20022975 PMC2820677

[B18] Chung D Kuan PF Li B Sanalkumar R Liang K Bresnick EH Discovering transcription factor binding sites in highly repetitive regions of genomes with multi-read analysis of ChIP-Seq data PLoS Comput Biol 2011 7 7 e1002111 10.1371/journal.pcbi.1002111 21779159 PMC3136429

[B19] Lander ES Linton LM Birren B Nusbaum C Zody MC Baldwin J Initial sequencing and analysis of the human genome Nature 2001 409 6822 860 921 10.1038/35057062 11237011

[B20] 1000 Genomes Project Consortium Abecasis GR Auton A Brooks LD DePristo MA Durbin RM An integrated map of genetic variation from 1,092 human genomes Nature 2012 491 7422 56 65 10.1038/nature11632 23128226 PMC3498066

[B21] Hangauer MJ Vaughn IW McManus MT Pervasive transcription of the human genome produces thousands of previously unidentified long intergenic noncoding RNAs PLoS Genet 2013 9 6 e1003569 10.1371/journal.pgen.1003569 23818866 PMC3688513

[B22] Pickrell JK Gaffney DJ Gilad Y Pritchard JK False positive peaks in ChIP-seq and other sequencing-based functional assays caused by unannotated high copy number regions Bioinformatics 2011 27 15 2144 2146 10.1093/bioinformatics/btr354 21690102 PMC3137225

[B23] Medvedev P Stanciu M Brudno M Computational methods for discovering structural variation with next-generation sequencing Nat Methods 2009 6 11 S13 S20 19844226 10.1038/nmeth.1374

[B24] Lee H Popodi E Foster PL Tang H Detection of structural variants involving repetitive regions in the reference genome J Comput Biol 2014 21 3 219 233 10.1089/cmb.2013.0129 24552580

[B25] Schmieder R Edwards R Quality control and preprocessing of metagenomic datasets Bioinformatics 2011 27 6 863 864 10.1093/bioinformatics/btr026 21278185 PMC3051327

[B26] Olson LE Soriano P Increased PDGFRalpha activation disrupts connective tissue development and drives systemic fibrosis Dev Cell 2009 16 2 303 313 10.1016/j.devcel.2008.12.003 19217431 PMC2664622

[B27] Bolger AM Lohse M Usadel B Trimmomatic: A flexible trimmer for Illumina Sequence Data Bioinformatics 2014 30 15 2114 2120 10.1093/bioinformatics/btu170 24695404 PMC4103590

[B28] Chen N Andreas D Baxevanis Using RepeatMasker to identify repetitive elements in genomic sequences Curr Protoc Bioinformatics 2004 Chapter 4:Unit 4.10 10.1002/0471250953.bi0410s05 18428725

[B29] RepeatMasker Open-3.0 http://www.repeatmasker.org

[B30] FAQ BED format http://genome.ucsc.edu/goldenPath/help/customTrack.html BED

[B31] Trapnell C Roberts A Goff L Pertea G Kim D Kelley DR Differential gene and transcript expression analysis of RNA-seq experiments with TopHat and Cufflinks Nat Protoc 2012 7 3 562 578 10.1038/nprot.2012.016 22383036 PMC3334321

[B32] Team RDC R: A Language and Environment for Statistical Computing 2013

[B33] Langmead B Trapnell C Pop M Salzberg SL Ultrafast and memory-efficient alignment of short DNA sequences to the human genome Genome Biol 2009 10 3 R25 10.1186/gb-2009-10-3-r25 19261174 PMC2690996

[B34] Feng J Liu T Zhang Y Using MACS to identify peaks from ChIP-Seq data Curr Protoc Bioinformatics 2011 Chapter 2:Unit 2.14 10.1002/0471250953.bi0214s34 PMC3120977 21633945

[B35] Machanick P Bailey TL MEME-ChIP: motif analysis of large DNA datasets Bioinformatics 2011 27 12 1696 1697 10.1093/bioinformatics/btr189 21486936 PMC3106185

[B36] Rosenbloom KR Sloan CA Malladi VS Dreszer TR Learned K Kirkup VM ENCODE data in the UCSC Genome Browser: year 5 update Nucleic Acids Res 2013 41 Database issue D56 D63 23193274 10.1093/nar/gks1172 PMC3531152

