# Supplementary material for: Detrimental effects of duplicate reads and low complexity regions on RNA- and ChIP-seq data
Source: BMC Bioinformatics. 2015 Sep 25;16(Suppl 13):S10. doi: 10.1186/1471-2105-16-S13-S10 (PMC4597324; doi:10.1186/1471-2105-16-S13-S10)
Supplement: Additional File 7 — Investigation of the biology of genes lost after filtering out low complexity reads with RepeatSoaker. [file 1471-2105-16-S13-S10-S7.pdf]

RepeatSoaker effect on DEGs detection

Using adapter trimmed, duplicates removed mouse RNA-seq data, we are investigating the biology of genes removed by RepeatSoaker. That is, What are the properties of the genes lost when removing reads overlapping low complexity regions.

First, we explore overlap among gene lists without RepeatSoaker treatment (remDup_all) and with RepeatSoaker treatment at different thresholds (remDup_75 - remDup_0)

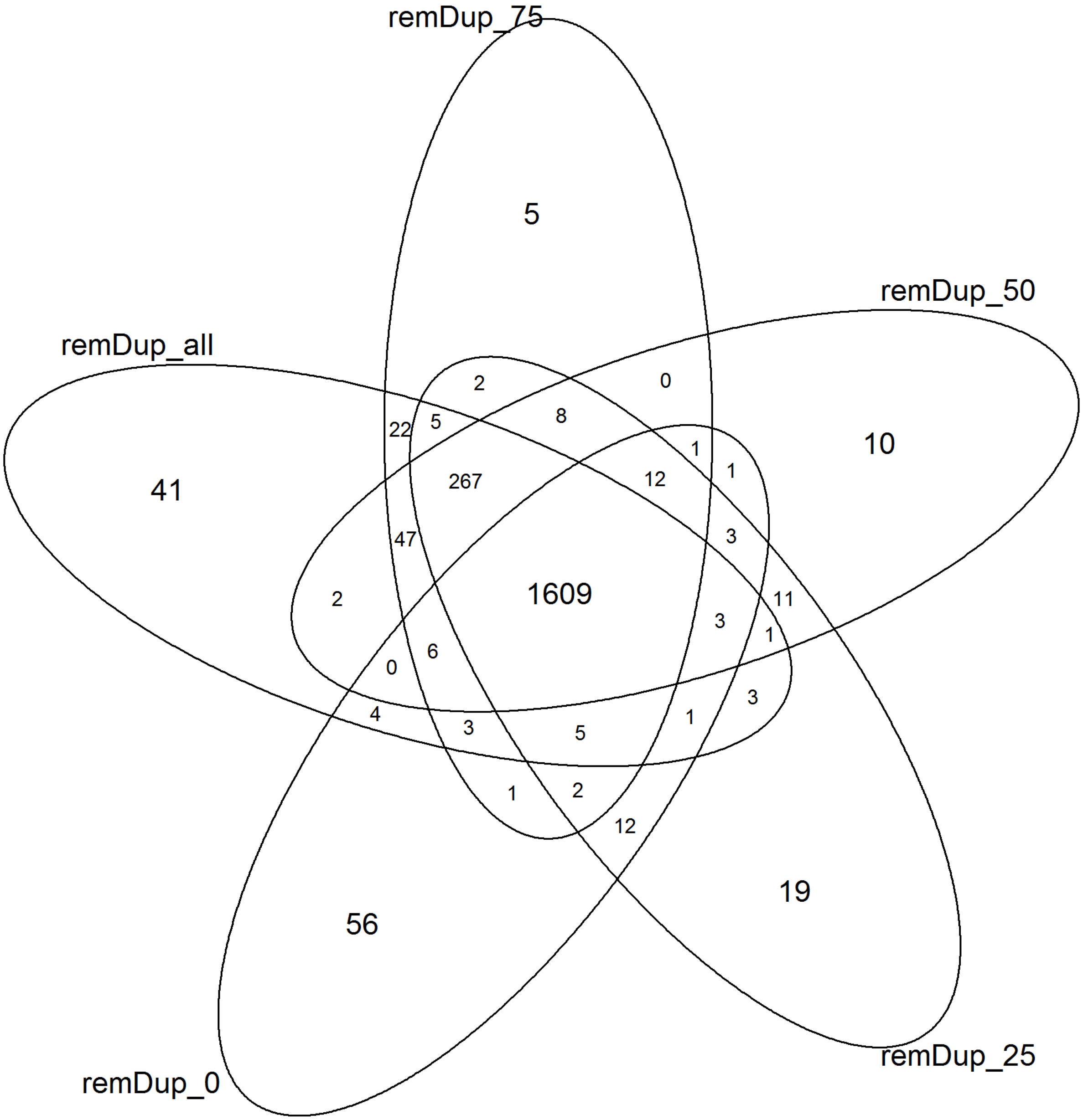

We observe that “RepeatSoaking” diminishes the number of differentially expressed genes. More rigorous soaking (less strict overlap threshold) also increases the number of genes not detected previously (the numbers in the “leaves” of the Venn diagram).

Withour RepeatSoaker, we have (all genes in the remDup_All oval):

```
## Number of differentially expressed PROBES, no
RepeatSoaker: 2019
## Number of differentially expressed GENES, no
RepeatSoaker: 1991
```

Some probes map to multiple genes, hence the discrepancy in counts of probes and genes.

After trimming the data with 0% RepeatSoaker settings, we have (remDup_0 oval):

```
## Number of differentially expressed PROBES, with
RepeatSoaker: 1719
## Number of differentially expressed GENES, with
RepeatSoaker: 1697
```

Genes without vs. Genes with RepeatSoaker comparison

We compare enrichment analyses results using genes with and without reads overlapping low complexity regions (remDup_all vs. remDup_0).

KEGG enrichment analysis without RepeatSoaker

```
##
## KEGG.db contains mappings based on older data because
the original resource was removed from the the public
##   domain before the most recent update was produced.
This package should now be considered deprecated and
##   future versions of Bioconductor may not have it
available.  Users who want more current data are
##   encouraged to look at the KEGGREST or reactome.db
packages
##
## The number of enriched KEGG :35
```

	KEGGID	Pvalue	OddsRatio	ExpCount	Count	Size	Term	p.adj
1	03010	7.170e-43	15.319	12.275	73	119	Ribosome	2.509e-41
2	04510	1.069e-05	2.321	20.631	41	200	Focal adhesion	1.872e-04
3	04141	8.979e-05	2.252	17.433	34	169	Protein processing in endoplasmic reticulum	1.048e-03
4	04612	2.666e-04	2.851	8.046	19	78	Antigen processing and presentation	2.333e-03
5	04916	1.095e-03	2.352	10.315	21	100	Melanogenesis	7.664e-03
6	04115	1.657e-03	2.614	7.221	16	70	p53 signaling pathway	9.666e-03
7	05200	2.245e-03	1.626	33.525	50	325	Pathways in cancer	1.122e-02
8	00330	2.710e-03	2.791	5.570	13	54	Arginine and proline metabolism	1.185e-02
9	05414	3.729e-03	2.237	9.181	18	89	Dilated cardiomyopathy	1.450e-02
10	04144	5.795e-03	1.679	22.694	35	220	Endocytosis	1.859e-02
11	05218	5.842e-03	2.317	7.427	15	72	Melanoma	1.859e-02
12	05215	8.602e-03	2.080	9.181	17	89	Prostate cancer	2.466e-02
13	05410	9.731e-03	2.102	8.562	16	83	Hypertrophic cardiomyopathy (HCM)	2.466e-02
14	04614	9.864e-03	4.039	1.960	6	19	Renin-angiotensin system	2.466e-02
15	05211	1.240e-02	2.159	7.324	14	71	Renal cell carcinoma	2.893e-02
16	00920	1.418e-02	5.824	1.032	4	10	Sulfur metabolism	3.102e-02
17	05222	1.513e-02	1.982	8.974	16	87	Small cell lung cancer	3.115e-02
18	04150	1.750e-02	2.298	5.467	11	53	mTOR signaling pathway	3.402e-02
19	00030	2.069e-02	2.918	2.888	7	28	Pentose phosphate pathway	3.811e-02
20	03013	2.223e-02	1.615	17.330	26	168	RNA transport	3.890e-02

KEGG enrichment analysis with RepeatSoaker

```
## The number of enriched KEGG :33
```

	KEGGID	Pvalue	OddsRatio	ExpCount	Count	Size	Term	p.adj
1	03010	3.754e-41	15.122	10.685	68	119	Ribosome	1.239e-39
2	04510	8.150e-07	2.644	17.958	40	200	Focal adhesion	1.345e-05
3	04612	1.150e-05	3.582	7.004	20	78	Antigen processing and presentation	1.265e-04
4	04141	3.335e-05	2.445	15.174	32	169	Protein processing in endoplasmic reticulum	2.751e-04
5	04916	4.723e-04	2.587	8.979	20	100	Melanogenesis	3.118e-03
6	05200	1.973e-03	1.680	29.182	45	325	Pathways in cancer	9.961e-03
7	04150	2.113e-03	3.007	4.759	12	53	mTOR signaling pathway	9.961e-03
8	04614	5.009e-03	4.716	1.706	6	19	Renin-angiotensin system	1.720e-02
9	05414	5.212e-03	2.255	7.991	16	89	Dilated cardiomyopathy	1.720e-02
10	05215	5.212e-03	2.255	7.991	16	89	Prostate cancer	1.720e-02
11	05410	6.396e-03	2.268	7.453	15	83	Hypertrophic cardiomyopathy (HCM)	1.919e-02
12	04144	7.369e-03	1.699	19.754	31	220	Endocytosis	2.026e-02
13	04020	8.579e-03	1.767	15.983	26	178	Calcium signaling pathway	2.178e-02
14	04910	1.017e-02	1.866	12.301	21	137	Insulin signaling pathway	2.383e-02
15	05218	1.083e-02	2.261	6.465	13	72	Melanoma	2.383e-02
16	04360	1.248e-02	1.855	11.762	20	131	Axon guidance	2.574e-02
17	03013	1.464e-02	1.718	15.085	24	168	RNA transport	2.843e-02
18	04970	1.859e-02	2.083	6.914	13	77	Salivary secretion	2.923e-02
19	04512	2.011e-02	1.994	7.722	14	86	ECM-receptor interaction	2.923e-02
20	00330	2.026e-02	2.326	4.849	10	54	Arginine and proline metabolism	2.923e-02

GO enrichment analysis without RepeatSoaker

```
## The number of enriched GO :2035
```

	GOBPID	Pvalue	OddsRatio	ExpCount	Count	Size	Term	p.adj
1	GO:0008152	3.949e-69	2.348	714.5	1074	9209	metabolic process	8.037e-66
2	GO:0044237	1.116e-68	2.358	601.0	951	7747	cellular metabolic process	1.135e-65
3	GO:0044238	2.303e-68	2.349	615.5	966	7933	primary metabolic process	1.562e-65
4	GO:0071704	2.298e-67	2.328	644.6	995	8309	organic substance metabolic process	1.169e-64
5	GO:0009987	8.617e-61	2.353	1055.6	1384	13606	cellular process	3.507e-58
6	GO:0043170	9.411e-56	2.200	516.3	820	6655	macromolecule metabolic process	3.192e-53
7	GO:0044260	1.036e-54	2.228	450.5	741	5807	cellular macromolecule metabolic process	3.012e-52
8	GO:0071840	1.447e-43	2.203	308.6	537	3978	cellular component organization or biogenesis	3.682e-41
9	GO:0019222	1.719e-41	2.087	366.0	601	4717	regulation of metabolic process	3.886e-39
10	GO:1901576	8.104e-41	2.111	336.3	563	4335	organic substance biosynthetic process	1.649e-38
11	GO:0044249	9.538e-41	2.118	330.0	555	4253	cellular biosynthetic process	1.765e-38
12	GO:0016043	1.126e-40	2.163	298.6	516	3849	cellular component organization	1.909e-38
13	GO:0009058	1.707e-40	2.097	342.1	569	4409	biosynthetic process	2.672e-38
14	GO:0031323	7.132e-40	2.125	313.4	532	4040	regulation of cellular metabolic process	1.037e-37
15	GO:0010467	4.121e-39	2.126	303.2	517	3908	gene expression	5.591e-37
16	GO:1901360	1.029e-38	2.030	373.3	601	4812	organic cyclic compound metabolic process	1.274e-36
17	GO:0034645	1.064e-38	2.173	270.7	475	3489	cellular macromolecule biosynthetic process	1.274e-36
18	GO:0009059	2.173e-38	2.154	277.8	483	3580	macromolecule biosynthetic process	2.457e-36
19	GO:0006807	1.134e-37	1.994	390.6	618	5034	nitrogen compound metabolic process	1.214e-35
20	GO:0019538	1.286e-37	2.120	288.4	494	3717	protein metabolic process	1.308e-35

GO enrichment analysis with RepeatSoaker

```
## The number of enriched GO :2025
```

	GOBPID	Pvalue	OddsRatio	ExpCount	Count	Size	Term	p.adj
1	GO:0008152	1.189e-59	2.345	607.7	917	9209	metabolic process	1.782e-56
2	GO:0044237	1.760e-59	2.357	511.2	813	7747	cellular metabolic process	1.782e-56
3	GO:0044238	6.221e-59	2.343	523.5	825	7933	primary metabolic process	4.199e-56
4	GO:0071704	1.769e-57	2.312	548.3	848	8309	organic substance metabolic process	8.958e-55
5	GO:0009987	2.403e-54	2.396	897.8	1185	13606	cellular process	9.733e-52
6	GO:0043170	5.039e-50	2.228	439.2	706	6655	macromolecule metabolic process	1.701e-47
7	GO:0044260	1.316e-49	2.265	383.2	640	5807	cellular macromolecule metabolic process	3.807e-47
8	GO:1901576	1.403e-40	2.214	286.1	497	4335	organic substance biosynthetic process	3.550e-38
9	GO:0034645	4.446e-40	2.317	230.2	425	3489	cellular macromolecule biosynthetic process	1.000e-37
10	GO:0071840	5.087e-40	2.245	262.5	466	3978	cellular component organization or biogenesis	1.030e-37
11	GO:0009059	8.307e-40	2.296	236.2	432	3580	macromolecule biosynthetic process	1.529e-37
12	GO:0019222	1.352e-39	2.156	311.3	525	4717	regulation of metabolic process	2.282e-37
13	GO:0009058	1.538e-39	2.185	290.9	500	4409	biosynthetic process	2.396e-37
14	GO:0044249	1.929e-39	2.200	280.6	487	4253	cellular biosynthetic process	2.790e-37
15	GO:0010467	2.978e-39	2.237	257.9	458	3908	gene expression	4.020e-37
16	GO:0031323	6.336e-39	2.213	266.6	468	4040	regulation of cellular metabolic process	8.019e-37
17	GO:0016043	1.768e-37	2.206	254.0	448	3849	cellular component organization	2.105e-35
18	GO:1901360	4.197e-37	2.098	317.5	525	4812	organic cyclic compound metabolic process	4.722e-35
19	GO:0080090	2.536e-36	2.162	263.9	457	3999	regulation of primary metabolic process	2.702e-34
20	GO:0006807	5.539e-36	2.058	332.2	539	5034	nitrogen compound metabolic process	5.609e-34

Reactome enrichment analysis without RepeatSoaker

```
## The number of enriched Reactome pathways:403
```

PathwayID	Pathway	N.Selected	N.In.Category	p.value	p.adj
4809935	Mus musculus: Gene Expression	138	839	1.706e-47	1.810e-
4809951	Mus musculus: Metabolism of proteins	123	691	1.617e-45	8.581e-
4809858	Mus musculus: Metabolism	172	1504	6.770e-40	2.394e-
4810524	Mus musculus: Metabolism of RNA	83	384	2.675e-36	7.097e-
4810844	Mus musculus: Metabolism of mRNA	80	360	9.222e-36	1.957e-
4809947	Mus musculus: GTP hydrolysis and joining of the 60S ribosomal subunit	68	249	3.095e-35	5.473e-
4810940	Mus musculus: Nonsense-Mediated Decay	67	245	9.088e-35	1.156e-
4810941	Mus musculus: Nonsense Mediated Decay Enhanced by the Exon Junction Complex	67	245	9.088e-35	1.156e-
4809948	Mus musculus: Cap-dependent Translation Initiation	68	256	1.311e-34	1.156e-
4809949	Mus musculus: Eukaryotic Translation Initiation	68	256	1.311e-34	1.156e-
4810290	Mus musculus: L13a-mediated translational silencing of Ceruloplasmin expression	67	247	1.379e-34	1.156e-
4810291	Mus musculus: 3' -UTR-mediated translational regulation	67	247	1.379e-34	1.156e-
4810293	Mus musculus: Eukaryotic Translation Elongation	65	229	1.416e-34	1.156e-
4810292	Mus musculus: Peptide chain elongation	64	225	4.141e-34	3.138e-
4809956	Mus musculus: Formation of a pool of free 40S subunits	65	235	5.134e-34	3.631e-
4810939	Mus musculus: Nonsense Mediated Decay Independent of the Exon Junction Complex	64	228	7.928e-34	5.257e-
4810283	Mus musculus: Eukaryotic Translation Termination	63	224	2.320e-33	1.448e-
4809950	Mus musculus: Translation	70	297	8.212e-33	4.840e-
4811072	Mus musculus: SRP-dependent cotranslational protein targeting to membrane	63	254	9.906e-31	5.531e-
4810016	Mus musculus: Immune System	108	1045	3.316e-22	1.759e-

Reactome enrichment analysis with RepeatSoaker

```
## The number of enriched Reactome pathways:390
```

PathwayID	Pathway	N.Selected	N.In.Category	p.value	p.adj
4809951	Mus musculus: Metabolism of proteins	113	691	3.822e-45	3.871e-
4809935	Mus musculus: Gene Expression	123	839	1.142e-44	5.784e-
4810524	Mus musculus: Metabolism of RNA	78	384	4.095e-37	1.383e-
4809947	Mus musculus: GTP hydrolysis and joining of the 60S ribosomal subunit	65	249	1.162e-36	2.944e-
4810844	Mus musculus: Metabolism of mRNA	75	360	2.258e-36	4.575e-
4810940	Mus musculus: Nonsense-Mediated Decay	64	245	3.931e-36	5.364e-
4810941	Mus musculus: Nonsense Mediated Decay Enhanced by the Exon Junction Complex	64	245	3.931e-36	5.364e-
4809948	Mus musculus: Cap-dependent Translation Initiation	65	256	4.765e-36	5.364e-
4809949	Mus musculus: Eukaryotic Translation Initiation	65	256	4.765e-36	5.364e-
4810290	Mus musculus: L13a-mediated translational silencing of Ceruloplasmin expression	64	247	5.908e-36	5.441e-
4810291	Mus musculus: 3' -UTR-mediated translational regulation	64	247	5.908e-36	5.441e-
4810293	Mus musculus: Eukaryotic Translation Elongation	62	229	8.482e-36	7.160e-
4810292	Mus musculus: Peptide chain elongation	61	225	2.860e-35	2.154e-
4809956	Mus musculus: Formation of a pool of free 40S subunits	62	235	2.977e-35	2.154e-
4810939	Mus musculus: Nonsense Mediated Decay Independent of the Exon Junction Complex	61	228	5.385e-35	3.636e-
4809858	Mus musculus: Metabolism	147	1504	1.172e-34	7.417e-
4810283	Mus musculus: Eukaryotic Translation Termination	60	224	1.817e-34	1.082e-
4809950	Mus musculus: Translation	67	297	1.985e-34	1.117e-
4811072	Mus musculus: SRP-dependent cotranslational protein targeting to membrane	60	254	6.624e-32	3.532e-
4809952	Mus musculus: Ribosomal scanning and start codon recognition	34	124	2.032e-20	1.029e-

Genes unique for different RepeatSoaker settings

Now, we check what those genes unique to each RepeatSoaker % are (leaves of the Venn diagram). We will look at:

1) Gene names and their description. Note that not all probes can be mapped to gene names, and some probes map to the same gene - therefore, the numbers in the Venn diagram and the tables below differ.

2) GO, KEGG and Reactome Pathway enrichment of those genes, if any.

Unique genes without RepeatSoaker

18	9930104L06Rik	RIKEN cDNA 9930104L06 gene [Source:MGI Symbol;Acc:MGI:3041172]
20	Adarb2	adenosine deaminase, RNA-specific, B2 [Source:MGI Symbol;Acc:MGI:2151118]
16	Creg1	cellular repressor of E1A-stimulated genes 1 [Source:MGI Symbol;Acc:MGI:1344382]
29	Etohi1	ethanol induced 1 [Source:MGI Symbol;Acc:MGI:1261426]
22	Gm11541	predicted gene 11541 [Source:MGI Symbol;Acc:MGI:3650066]
31	Gm14295	predicted gene 14295 [Source:MGI Symbol;Acc:MGI:3709624]
34	Gm15564	predicted gene 15564 [Source:MGI Symbol;Acc:MGI:3783013]
32	Gm15663	predicted gene 15663 [Source:MGI Symbol;Acc:MGI:3783105]
35	Gm24187	predicted gene, 24187 [Source:MGI Symbol;Acc:MGI:5453964]
30	Gm24270	predicted gene, 24270 [Source:MGI Symbol;Acc:MGI:5454047]
25	Gm26920	predicted gene, 26920 [Source:MGI Symbol;Acc:MGI:5504035]
3	Gm872	RIKEN cDNA 4930485B16 gene [Source:MGI Symbol;Acc:MGI:1922208]
23	Gpr116	G protein-coupled receptor 116 [Source:MGI Symbol;Acc:MGI:2182928]
28	H2-Q6	histocompatibility 2, Q region locus 6 [Source:MGI Symbol;Acc:MGI:95935]
11	Ing3	inhibitor of growth family, member 3 [Source:MGI Symbol;Acc:MGI:1919027]
10	Klhl30	kelch-like 30 [Source:MGI Symbol;Acc:MGI:1918038]
21	Lins	lines homolog (Drosophila) [Source:MGI Symbol;Acc:MGI:1919885]
24	Mex3b	mex3 homolog B (C. elegans) [Source:MGI Symbol;Acc:MGI:1918252]
27	Nanos1	nanos homolog 1 (Drosophila) [Source:MGI Symbol;Acc:MGI:2669254]
9	Ndufb8	NADH dehydrogenase (ubiquinone) 1 beta subcomplex 8 [Source:MGI Symbol;Acc:MGI:1914514]
1	Npepps	aminopeptidase puromycin sensitive [Source:MGI Symbol;Acc:MGI:1101358]
15	Parp11	poly (ADP-ribose) polymerase family, member 11 [Source:MGI Symbol;Acc:MGI:2141505]
2	Ppil4	peptidylprolyl isomerase (cyclophilin)-like 4 [Source:MGI Symbol;Acc:MGI:1914668]
6	Rbm26	RNA binding motif protein 26 [Source:MGI Symbol;Acc:MGI:1921463]
13	Rspo4	R-spondin family, member 4 [Source:MGI Symbol;Acc:MGI:1924467]
26	Sat2	spermidine/spermine N1-acetyl transferase 2 [Source:MGI Symbol;Acc:MGI:1916465]
4	Shmt1	serine hydroxymethyltransferase 1 (soluble) [Source:MGI Symbol;Acc:MGI:98299]
8	Slc4a8	solute carrier family 4 (anion exchanger), member 8 [Source:MGI Symbol;Acc:MGI:1928745]
19	Tnfrsf26	tumor necrosis factor receptor superfamily, member 26 [Source:MGI Symbol;Acc:MGI:2651928]
14	Tstd2	thiosulfate sulfurtransferase (rhodanese)-like domain containing 2 [Source:MGI Symbol;Acc:MGI:3039624]
7	Utp23	UTP23, small subunit (SSU) processome component, homolog (yeast) [Source:MGI Symbol;Acc:MGI:1925831]

```
## The number of enriched KEGG :10
```

	KEGGID	Pvalue	OddsRatio	ExpCount	Count	Size	Term	p.adj
1	00460	0.00360	443.40	0.003604	1	6	Cyanoamino acid metabolism	0.03600
2	00670	0.01137	122.93	0.011411	1	19	One carbon pool by folate	0.04372
3	01100	0.01944	13.91	0.711111	3	1184	Metabolic pathways	0.04372
4	00260	0.02027	66.90	0.020420	1	34	Glycine, serine and threonine metabolism	0.04372
5	05330	0.03087	43.17	0.031231	1	52	Allograft rejection	0.04372
6	00330	0.03205	41.53	0.032432	1	54	Arginine and proline metabolism	0.04372
7	05332	0.03205	41.53	0.032432	1	54	Graft-versus-host disease	0.04372
8	04940	0.03498	37.92	0.035435	1	59	Type I diabetes mellitus	0.04372
9	05320	0.03965	33.28	0.040240	1	67	Autoimmune thyroid disease	0.04405
10	04612	0.04604	28.48	0.046847	1	78	Antigen processing and presentation	0.04604

```
## The number of enriched GO :31
```

	GOBPID	Pvalue	OddsRatio	ExpCount	Count	Size	Term	p.adj
1	GO:0019264	0.002585	798.07	0.002586	1	2	glycine biosynthetic process from serine	0.03373
2	GO:0046655	0.002585	798.07	0.002586	1	2	folic acid metabolic process	0.03373
3	GO:0006565	0.003874	399.02	0.003879	1	3	L-serine catabolic process	0.03373
4	GO:0006545	0.006449	199.49	0.006465	1	5	glycine biosynthetic process	0.03373
5	GO:0008215	0.007734	159.59	0.007758	1	6	spermine metabolic process	0.03373
6	GO:0009113	0.007734	159.59	0.007758	1	6	purine nucleobase biosynthetic process	0.03373
7	GO:0006120	0.009018	132.98	0.009051	1	7	mitochondrial electron transport, NADH to ubiquinone	0.03373
8	GO:0006563	0.010299	113.98	0.010345	1	8	L-serine metabolic process	0.03373
9	GO:0008216	0.010299	113.98	0.010345	1	8	spermidine metabolic process	0.03373
10	GO:0046112	0.011580	99.73	0.011638	1	9	nucleobase biosynthetic process	0.03373
11	GO:0009070	0.015410	72.52	0.015517	1	12	serine family amino acid biosynthetic process	0.03373
12	GO:0043968	0.015410	72.52	0.015517	1	12	histone H2A acetylation	0.03373
13	GO:0006144	0.016684	66.47	0.016810	1	13	purine nucleobase metabolic process	0.03373
14	GO:0043129	0.016684	66.47	0.016810	1	13	surfactant homeostasis	0.03373
15	GO:0015980	0.017920	10.45	0.205598	2	159	energy derivation by oxidation of organic compounds	0.03373
16	GO:0048875	0.019227	56.97	0.019396	1	15	chemical homeostasis within a tissue	0.03373
17	GO:0009071	0.020496	53.17	0.020689	1	16	serine family amino acid catabolic process	0.03373
18	GO:0042775	0.020496	53.17	0.020689	1	16	mitochondrial ATP synthesis coupled electron transport	0.03373
19	GO:0006544	0.021763	49.85	0.021982	1	17	glycine metabolic process	0.03373
20	GO:0006595	0.021763	49.85	0.021982	1	17	polyamine metabolic process	0.03373

```
## The number of enriched Reactome pathways:0
```

Unique genes with 75% RepeatSoaker

	external_gene_id	description
5	Cyb5d2	cytochrome b5 domain containing 2 [Source:MGI Symbol;Acc:MGI:2684848]
2	Hspbap1	Hspb associated protein 1 [Source:MGI Symbol;Acc:MGI:1913917]
1	Mpp5	membrane protein, palmitoylated 5 (MAGUK p55 subfamily member 5) [Source:MGI Symbol;Acc:MGI:1927339]
4	Mypop	Myb-related transcription factor, partner of profilin [Source:MGI Symbol;Acc:MGI:2446472]
3	Tead2	TEA domain family member 2 [Source:MGI Symbol;Acc:MGI:104904]

```
## The number of enriched KEGG :1
```

	KEGGID	Pvalue	OddsRatio	ExpCount	Count	Size	Term	p.adj
1	04530	0.02057	Inf	0.02057	1	137	Tight junction	0.02057

```
## The number of enriched GO :52
```

	GOBPID	Pvalue	OddsRatio	ExpCount	Count	Size	Term	p.adj
1	GO:0035750	0.0002086	Inf	0.0002086	1	1	protein localization to myelin sheath abaxonal region	0.01085
2	GO:0032287	0.0014592	998.46	0.0014599	1	7	peripheral nervous system myelin maintenance	0.01856
3	GO:0043217	0.0022922	598.98	0.0022942	1	11	myelin maintenance	0.01856
4	GO:0007399	0.0024296	21.79	0.3226412	3	1547	nervous system development	0.01856
5	GO:0008105	0.0025004	544.50	0.0025027	1	12	asymmetric protein localization	0.01856
6	GO:0048368	0.0029167	460.69	0.0029198	1	14	lateral mesoderm development	0.01856
7	GO:0030903	0.0035408	374.27	0.0035455	1	17	notochord development	0.01856
8	GO:0022011	0.0037487	352.24	0.0037541	1	18	myelination in peripheral nervous system	0.01856
9	GO:0032288	0.0037487	352.24	0.0037541	1	18	myelin assembly	0.01856
10	GO:0032292	0.0037487	352.24	0.0037541	1	18	peripheral nervous system axon ensheathment	0.01856
11	GO:0048339	0.0041646	315.13	0.0041712	1	20	paraxial mesoderm development	0.01856
12	GO:0014044	0.0043724	299.36	0.0043797	1	21	Schwann cell development	0.01856
13	GO:0035329	0.0047881	272.12	0.0047969	1	23	hippo signaling cascade	0.01856
14	GO:0046939	0.0049958	260.28	0.0050054	1	24	nucleotide phosphorylation	0.01856
15	GO:0014037	0.0054112	239.44	0.0054225	1	26	Schwann cell differentiation	0.01876
16	GO:0003143	0.0116258	108.70	0.0116793	1	56	embryonic heart tube morphogenesis	0.03548
17	GO:0048646	0.0120655	17.87	0.1801952	2	864	anatomical structure formation involved in morphogenesis	0.03548
18	GO:0007422	0.0126586	99.62	0.0127221	1	61	peripheral nervous system development	0.03548
19	GO:0021782	0.0138967	90.54	0.0139735	1	67	glial cell development	0.03548
20	GO:0090002	0.0143092	87.87	0.0143906	1	69	establishment of protein localization to plasma membrane	0.03548

```
## The number of enriched Reactome pathways:6
```

	PathwayID	Pathway	N.Selected	N.In.Category	p.value	p.adj
6	4810822	Mus musculus: Tight junction interactions	1	10	0.000948	0.003620
7	4811094	Mus musculus: YAP1- and WWTR1 (TAZ)-stimulated gene expression	1	11	0.001034	0.003620
4	4810815	Mus musculus: Cell-cell junction organization	1	37	0.003272	0.007635
5	4810816	Mus musculus: Cell junction organization	1	63	0.005506	0.009635
3	4810638	Mus musculus: Cell-Cell communication	1	100	0.008678	0.012149
2	4810429	Mus musculus: Generic Transcription Pathway	1	128	0.011073	0.012918

Unique genes with 50% RepeatSoaker

	external_gene_id	description
7	Chrm2	cholinergic receptor, muscarinic 2, cardiac [Source:MGI Symbol;Acc:MGI:88397]
2	Ern1	endoplasmic reticulum (ER) to nucleus signalling 1 [Source:MGI Symbol;Acc:MGI:1930134]
4	Gnl1	guanine nucleotide binding protein-like 1 [Source:MGI Symbol;Acc:MGI:95764]
5	Gxylt1	glucoside xylosyltransferase 1 [Source:MGI Symbol;Acc:MGI:2684933]
9	Ism1	isthmin 1 homolog (zebrafish) [Source:MGI Symbol;Acc:MGI:2442963]
8	Lekr1	leucine, glutamate and lysine rich 1 [Source:MGI Symbol;Acc:MGI:3645902]
6	Mob1a	MOB kinase activator 1A [Source:MGI Symbol;Acc:MGI:2442631]
1	Slc29a3	solute carrier family 29 (nucleoside transporters), member 3 [Source:MGI Symbol;Acc:MGI:1918529]
3	Zfp346	zinc finger protein 346 [Source:MGI Symbol;Acc:MGI:1349417]

```
## The number of enriched KEGG :1
```

	KEGGID	Pvalue	OddsRatio	ExpCount	Count	Size	Term	p.adj
1	00514	0.01925	78.75	0.01937	1	43	Other types of O-glycan biosynthesis	0.01925

```
## The number of enriched GO :32
```

GOBPID	Pvalue	OddsRatio	ExpCount	Count	Size	Term	[illegible]
GO:0006987	0.001001	1711.71	0.001001	1	3	activation of signaling protein activity involved in unfolded protein response	0.
GO:0016266	0.001001	1711.71	0.001001	1	3	O-glycan processing	0.
GO:1900103	0.001334	1141.10	0.001335	1	4	positive regulation of endoplasmic reticulum unfolded protein response	0.
GO:0032075	0.001668	855.79	0.001668	1	5	positive regulation of nuclease activity	0.
GO:1900101	0.001668	855.79	0.001668	1	5	regulation of endoplasmic reticulum unfolded protein response	0.
GO:0007213	0.002667	488.96	0.002670	1	8	G-protein coupled acetylcholine receptor signaling pathway	0.
GO:0032069	0.003333	380.27	0.003337	1	10	regulation of nuclease activity	0.
GO:0015858	0.004330	285.17	0.004338	1	13	nucleoside transport	0.
GO:0035329	0.007650	155.48	0.007675	1	23	hippo signaling cascade	0.
GO:0070059	0.009307	126.66	0.009343	1	28	intrinsic apoptotic signaling pathway in response to endoplasmic reticulum stress	0.
GO:0006493	0.009638	122.13	0.009677	1	29	protein O-linked glycosylation	0.
GO:0030968	0.010299	113.98	0.010345	1	31	endoplasmic reticulum unfolded protein response	0.
GO:0034620	0.010630	110.30	0.010678	1	32	cellular response to unfolded protein	0.
GO:1901264	0.011952	97.68	0.012013	1	36	carbohydrate derivative transport	0.
GO:0035967	0.012282	94.96	0.012347	1	37	cellular response to topologically incorrect protein	0.
GO:0006984	0.014261	81.37	0.014349	1	43	ER-nucleus signaling pathway	0.
GO:0006940	0.015249	75.94	0.015350	1	46	regulation of smooth muscle contraction	0.
GO:0006986	0.020833	55.08	0.021023	1	63	response to unfolded protein	0.
GO:0034976	0.022798	50.21	0.023025	1	69	response to endoplasmic reticulum stress	0.
GO:0035966	0.022798	50.21	0.023025	1	69	response to topologically incorrect protein	0.

```
## The number of enriched Reactome pathways:25
```

ayID	Pathway	N.Selected	N.In.Category	p.value
'67	Mus musculus: Muscarinic acetylcholine receptors	1	5	0.000930
20	Mus musculus: Transport of nucleosides and free purine and pyrimidine bases across the plasma membrane	1	7	0.001240
02	Mus musculus: Signaling by Hippo	1	20	0.003254
86	Mus musculus: A tetrasaccharide linker sequence is required for GAG synthesis	1	25	0.004027
21	Mus musculus: Transport of vitamins, nucleosides, and related molecules	1	32	0.005109
'66	Mus musculus: Amine ligand-binding receptors	1	35	0.005572
'38	Mus musculus: Activation of Chaperones by IRE1alpha	1	46	0.007270
51	Mus musculus: Chondroitin sulfate/dermatan sulfate metabolism	1	46	0.007270
13	Mus musculus: Heparan sulfate/heparin (HS-GAG) metabolism	1	48	0.007578
'36	Mus musculus: Unfolded Protein Response	1	62	0.009734
57	Mus musculus: Glycosaminoglycan metabolism	1	110	0.017094
58	Mus musculus: MPS IX - Natowicz syndrome	1	110	0.017094
59	Mus musculus: Mucopolysaccharidoses	1	110	0.017094
60	Mus musculus: MPS IIIB - Sanfilippo syndrome B	1	110	0.017094
61	Mus musculus: MPS VI - Maroteaux-Lamy syndrome	1	110	0.017094
62	Mus musculus: MPS IV - Morquio syndrome A	1	110	0.017094
63	Mus musculus: MPS IIIC - Sanfilippo syndrome C	1	110	0.017094
64	Mus musculus: MPS VII - Sly syndrome	1	110	0.017094
65	Mus musculus: MPS II - Hunter syndrome	1	110	0.017094
66	Mus musculus: MPS I - Hurler syndrome	1	110	0.017094

Unique genes with 25% RepeatSoaker

	external_gene_id	description
15	Cacna1c	calcium channel, voltage-dependent, L type, alpha 1C subunit [Source:MGI Symbol;Acc:MGI:103013]
2	Cars	cysteinyl-tRNA synthetase [Source:MGI Symbol;Acc:MGI:1351477]
18	Casc4	cancer susceptibility candidate 4 [Source:MGI Symbol;Acc:MGI:2443129]
11	Dennd6a	DENN/MADD domain containing 6A [Source:MGI Symbol;Acc:MGI:2442980]
10	Fam102a	family with sequence similarity 102, member A [Source:MGI Symbol;Acc:MGI:2138935]
6	Fgd1	FYVE, RhoGEF and PH domain containing 1 [Source:MGI Symbol;Acc:MGI:104566]
4	Fos	FBJ osteosarcoma oncogene [Source:MGI Symbol;Acc:MGI:95574]
3	Glt8d2	glycosyltransferase 8 domain containing 2 [Source:MGI Symbol;Acc:MGI:1922032]
9	Hiatl1	hippocampus abundant transcript-like 1 [Source:MGI Symbol;Acc:MGI:1913881]
8	Inpp4b	inositol polyphosphate-4-phosphatase, type II [Source:MGI Symbol;Acc:MGI:2158925]
13	Mars2	methionine-tRNA synthetase 2 (mitochondrial) [Source:MGI Symbol;Acc:MGI:2444136]
19	Nbeal1	neurobeachin like 1 [Source:MGI Symbol;Acc:MGI:2444343]
16	Pgpep1	pyroglutamyl-peptidase I [Source:MGI Symbol;Acc:MGI:1913772]
12	Phlpp1	PH domain and leucine rich repeat protein phosphatase 1 [Source:MGI Symbol;Acc:MGI:2138327]
1	Pknox1	Pbx/knotted 1 homeobox [Source:MGI Symbol;Acc:MGI:1201409]
5	Sdf2l1	stromal cell-derived factor 2-like 1 [Source:MGI Symbol;Acc:MGI:2149842]
7	Stx2	syntaxin 2 [Source:MGI Symbol;Acc:MGI:108059]
14	Usp14	ubiquitin specific peptidase 14 [Source:MGI Symbol;Acc:MGI:1928898]
17	Zfp462	zinc finger protein 462 [Source:MGI Symbol;Acc:MGI:107690]

```
## The number of enriched KEGG :4
```

	KEGGID	Pvalue	OddsRatio	ExpCount	Count	Size	Term	p.adj
1	00970	0.001909	41.841	0.06832	2	65	Aminoacyl-tRNA biosynthesis	0.007634
2	00450	0.017740	69.135	0.01787	1	17	Selenocompound metabolism	0.035479
3	04010	0.029632	9.605	0.28168	2	268	MAPK signaling pathway	0.036228
4	04130	0.036228	32.446	0.03679	1	35	SNARE interactions in vesicular transport	0.036228

```
## The number of enriched GO :101
```

	GOBPID	Pvalue	OddsRatio	ExpCount	Count	Size	Term	p.adj
1	GO:0006418	0.0004790	72.14	0.032494	2	41	tRNA aminoacylation for protein translation	0.01600
2	GO:0043038	0.0005519	66.98	0.034871	2	44	amino acid activation	0.01600
3	GO:0043039	0.0005519	66.98	0.034871	2	44	tRNA aminoacylation	0.01600
4	GO:0045670	0.0007413	57.40	0.040419	2	51	regulation of osteoclast differentiation	0.01600
5	GO:0017156	0.0008005	55.14	0.042004	2	53	calcium ion-dependent exocytosis	0.01600
6	GO:0030099	0.0013433	15.97	0.222700	3	281	myeloid cell differentiation	0.01600
7	GO:0030316	0.0013916	41.33	0.055477	2	70	osteoclast differentiation	0.01600
8	GO:0006423	0.0015845	1330.78	0.001585	1	2	cysteinyl-tRNA aminoacylation	0.01600
9	GO:0006431	0.0015845	1330.78	0.001585	1	2	methionyl-tRNA aminoacylation	0.01600
10	GO:0090038	0.0015845	1330.78	0.001585	1	2	negative regulation of protein kinase C signaling cascade	0.01600
11	GO:2000049	0.0023758	665.36	0.002378	1	3	positive regulation of cell-cell adhesion mediated by cadherin	0.01976
12	GO:0002761	0.0023873	31.20	0.072912	2	92	regulation of myeloid leukocyte differentiation	0.01976
13	GO:0051896	0.0025428	30.19	0.075290	2	95	regulation of protein kinase B signaling cascade	0.01976
14	GO:0002521	0.0031003	11.82	0.298782	3	377	leukocyte differentiation	0.02132
15	GO:0060083	0.0031665	443.56	0.003170	1	4	smooth muscle contraction involved in micturition	0.02132
16	GO:0043491	0.0038247	24.39	0.092725	2	117	protein kinase B signaling cascade	0.02220
17	GO:0000920	0.0039567	332.65	0.003963	1	5	cytokinetic cell separation	0.02220
18	GO:2000047	0.0039567	332.65	0.003963	1	5	regulation of cell-cell adhesion mediated by cadherin	0.02220
19	GO:0006399	0.0042169	23.17	0.097481	2	123	tRNA metabolic process	0.02242
20	GO:0036092	0.0047462	266.11	0.004755	1	6	phosphatidylinositol-3-phosphate biosynthetic process	0.02283

```
## The number of enriched Reactome pathways:51
```

	PathwayID	Pathway	N.Selected	N.In.Category	p.value	p.adj
8	4809934	Mus musculus: tRNA Aminoacylation	2	43	9.978e-05	0.008282
28	4810023	Mus musculus: Fc epsilon receptor (FCERI) signaling	2	164	1.348e-03	0.049909
75	4810594	Mus musculus: Negative regulation of the PI3K/AKT network	1	6	2.291e-03	0.049909
83	4811081	Mus musculus: Synthesis of IP2, IP, and Ins in the cytosol	1	10	3.597e-03	0.049909
13	4810001	Mus musculus: Signalling by NGF	2	279	3.787e-03	0.049909
59	4810383	Mus musculus: Activation of the AP-1 family of transcription factors	1	11	3.924e-03	0.049909
81	4811067	Mus musculus: Synthesis of PIPs at the early endosome membrane	1	12	4.250e-03	0.049909
77	4810727	Mus musculus: Mitochondrial tRNA aminoacylation	1	22	7.508e-03	0.049909
7	4809933	Mus musculus: Cytosolic tRNA aminoacylation	1	25	8.483e-03	0.049909
80	4811062	Mus musculus: Synthesis of PIPs at the plasma membrane	1	28	9.458e-03	0.049909
60	4810384	Mus musculus: MAPK targets/ Nuclear events mediated by MAP kinases	1	30	1.011e-02	0.049909
78	4810794	Mus musculus: Inhibition of Insulin Secretion by Adrenaline/Noradrenaline	1	30	1.011e-02	0.049909
76	4810711	Mus musculus: NCAM1 interactions	1	35	1.173e-02	0.049909
24	4810015	Mus musculus: Innate Immune System	2	519	1.244e-02	0.049909
35	4810145	Mus musculus: FCERI mediated MAPK activation	1	41	1.367e-02	0.049909
82	4811080	Mus musculus: Inositol phosphate metabolism	1	45	1.496e-02	0.049909
79	4811043	Mus musculus: PI Metabolism	1	46	1.529e-02	0.049909
65	4810549	Mus musculus: NRAGE signals death through JNK	1	47	1.561e-02	0.049909
36	4810150	Mus musculus: MAP kinase activation in TLR cascade	1	55	1.819e-02	0.049909
56	4810239	Mus musculus: G alpha (12/13) signalling events	1	57	1.883e-02	0.049909

Unique genes with 00% RepeatSoaker

21	Epha5	Eph receptor A5 [Source:MGI Symbol;Acc:MGI:99654]
29	Ephx1	epoxide hydrolase 1, microsomal [Source:MGI Symbol;Acc:MGI:95405]
19	Exo5	exonuclease 5 [Source:MGI Symbol;Acc:MGI:1920422]
9	Fbxl6	F-box and leucine-rich repeat protein 6 [Source:MGI Symbol;Acc:MGI:1354705]
27	Galt	galactose-1-phosphate uridyl transferase [Source:MGI Symbol;Acc:MGI:95638]
24	Gga1	golgi associated, gamma adaptin ear containing, ARF binding protein 1 [Source:MGI Symbol;Acc:MGI:2146207]
50	Gm13443	predicted gene 13443 [Source:MGI Symbol;Acc:MGI:3649615]
55	Gm5577	predicted gene 5577 [Source:MGI Symbol;Acc:MGI:3648213]
52	H2-Q1	histocompatibility 2, Q region locus 1 [Source:MGI Symbol;Acc:MGI:95928]
40	H2-Q7	histocompatibility 2, Q region locus 7 [Source:MGI Symbol;Acc:MGI:95936]
2	Hdgf	hepatoma-derived growth factor [Source:MGI Symbol;Acc:MGI:1194494]
42	Hs6st2	heparan sulfate 6-O-sulfotransferase 2 [Source:MGI Symbol;Acc:MGI:1354959]
12	Incenp	inner centromere protein [Source:MGI Symbol;Acc:MGI:1313288]
17	Invs	inversin [Source:MGI Symbol;Acc:MGI:1335082]
6	Ippk	inositol 1,3,4,5,6-pentakisphosphate 2-kinase [Source:MGI Symbol;Acc:MGI:1922928]
33	Mdga1	MAM domain containing glycosylphosphatidylinositol anchor 1 [Source:MGI Symbol;Acc:MGI:1922012]
45	Mocs1	molybdenum cofactor synthesis 1 [Source:MGI Symbol;Acc:MGI:1928904]
23	Nlrp3	NLR family, pyrin domain containing 3 [Source:MGI Symbol;Acc:MGI:2653833]
31	Nphp4	nephronophthisis 4 (juvenile) homolog (human) [Source:MGI Symbol;Acc:MGI:2384210]
15	Orc4	origin recognition complex, subunit 4 [Source:MGI Symbol;Acc:MGI:1347043]
48	Peg12	paternally expressed 12 [Source:MGI Symbol;Acc:MGI:1351637]
5	Pls3	plastin 3 (T-isoform) [Source:MGI Symbol;Acc:MGI:104807]
49	Pnmal2	PNMA-like 2 [Source:MGI Symbol;Acc:MGI:3645856]
39	Ppih	peptidyl prolyl isomerase H [Source:MGI Symbol;Acc:MGI:106499]
34	Prrt2	proline-rich transmembrane protein 2 [Source:MGI Symbol;Acc:MGI:1916267]
51	Rab11b	RAB11B, member RAS oncogene family [Source:MGI Symbol;Acc:MGI:99425]
53	Rpl13-ps1	ribosomal protein L13, pseudogene 1 [Source:MGI Symbol;Acc:MGI:3649805]
47	Rpl34-ps1	ribosomal protein L34, pseudogene 1 [Source:MGI Symbol;Acc:MGI:3704270]
44	Rpl7l1	ribosomal protein L7-like 1 [Source:MGI Symbol;Acc:MGI:1913479]
54	Rpsa-ps9	ribosomal protein SA, pseudogene 9 [Source:MGI Symbol;Acc:MGI:3650393]
7	Sap18	Sin3-associated polypeptide 18 [Source:MGI Symbol;Acc:MGI:1277978]
13	Sdc3	syndecan 3 [Source:MGI Symbol;Acc:MGI:1349163]

```
## The number of enriched KEGG :10
```

	KEGGID	Pvalue	OddsRatio	ExpCount	Count	Size	Term	p.adj
1	04962	0.007187	17.883	0.12913	2	43	Vasopressin-regulated water reabsorption	0.02646
2	04514	0.009462	7.849	0.44745	3	149	Cell adhesion molecules (CAMs)	0.02646
3	05330	0.010384	14.644	0.15616	2	52	Allograft rejection	0.02646
4	05332	0.011167	14.077	0.16216	2	54	Graft-versus-host disease	0.02646
5	04940	0.013232	12.832	0.17718	2	59	Type I diabetes mellitus	0.02646
6	05320	0.016856	11.239	0.20120	2	67	Autoimmune thyroid disease	0.02809
7	04612	0.022448	9.596	0.23423	2	78	Antigen processing and presentation	0.02965
8	04144	0.026769	5.223	0.66066	3	220	Endocytosis	0.02965
9	05416	0.028703	8.369	0.26727	2	89	Viral myocarditis	0.02965
10	04122	0.029647	38.778	0.03003	1	10	Sulfur relay system	0.02965

```
## The number of enriched GO :109
```

GOBPID	Pvalue	OddsRatio	ExpCount	Count	Size	Term	p.ad
GO:0002474	0.0007595	56.518	0.040878	2	20	antigen processing and presentation of peptide antigen via MHC class I	0.035
GO:0006258	0.0020439	Inf	0.002044	1	1	UDP-glucose catabolic process	0.035
GO:0052746	0.0020439	Inf	0.002044	1	1	inositol phosphorylation	0.035
GO:0048002	0.0027449	28.238	0.077667	2	38	antigen processing and presentation of peptide antigen	0.035
GO:0046483	0.0030615	2.451	9.401852	18	4600	heterocycle metabolic process	0.035
GO:0006725	0.0033522	2.427	9.477476	18	4637	cellular aromatic compound metabolic process	0.035
GO:0009227	0.0040837	498.417	0.004088	1	2	nucleotide-sugar catabolic process	0.035
GO:0036297	0.0040837	498.417	0.004088	1	2	interstrand cross-link repair	0.035
GO:0050713	0.0040837	498.417	0.004088	1	2	negative regulation of interleukin-1 beta secretion	0.035
GO:1900242	0.0040837	498.417	0.004088	1	2	regulation of synaptic vesicle endocytosis	0.035
GO:1901360	0.0050651	2.317	9.835155	18	4812	organic cyclic compound metabolic process	0.035
GO:0006011	0.0061194	249.198	0.006132	1	3	UDP-glucose metabolic process	0.035
GO:0007181	0.0061194	249.198	0.006132	1	3	transforming growth factor beta receptor complex assembly	0.035
GO:0044546	0.0061194	249.198	0.006132	1	3	NLRP3 inflammasome complex assembly	0.035
GO:0051961	0.0061194	249.198	0.006132	1	3	negative regulation of nervous system development	0.035
GO:0051964	0.0061194	249.198	0.006132	1	3	negative regulation of synapse assembly	0.035
GO:0060971	0.0061194	249.198	0.006132	1	3	embryonic heart tube left/right pattern formation	0.035
GO:0071918	0.0061194	249.198	0.006132	1	3	urea transmembrane transport	0.035
GO:2001135	0.0061194	249.198	0.006132	1	3	regulation of endocytic recycling	0.035
GO:0019882	0.0076101	16.378	0.130808	2	64	antigen processing and presentation	0.038

```
## The number of enriched Reactome pathways:43
```

PathwayID	Pathway	N.Selected	N.In.Category	p.value	p
4810988	Mus musculus: Endosomal/Vacuolar pathway	2	20	0.0002012	0.0
4810912	Mus musculus: HS-GAG biosynthesis	2	28	0.0003770	0.0
4810970	Mus musculus: Antigen Presentation: Folding, assembly and peptide loading of class I MHC	2	34	0.0005440	0.0
4809812	Mus musculus: Cell Cycle, Mitotic	4	398	0.0005849	0.0
4810913	Mus musculus: Heparan sulfate/heparin (HS-GAG) metabolism	2	48	0.0010488	0.0
4809813	Mus musculus: Cell Cycle	4	534	0.0017129	0.0
4809857	Mus musculus: Metabolism of carbohydrates	3	258	0.0019840	0.0
4810917	Mus musculus: Interferon gamma signaling	2	68	0.0020428	0.0
4809815	Mus musculus: Mitotic M-M/G1 phases	3	266	0.0021613	0.0
4810591	Mus musculus: Immunoregulatory interactions between a Lymphoid and a non-Lymphoid cell	2	78	0.0026568	0.0
4810985	Mus musculus: ER-Phagosome pathway	2	80	0.0027888	0.0
4809858	Mus musculus: Metabolism	6	1504	0.0029995	0.0
4810016	Mus musculus: Immune System	5	1045	0.0031610	0.0
4810986	Mus musculus: Antigen processing-Cross presentation	2	92	0.0036439	0.0
4810261	Mus musculus: Activation of BIM and translocation to mitochondria	1	4	0.0047290	0.0
4811082	Mus musculus: Synthesis of IPs in the nucleus	1	4	0.0047290	0.0
4810457	Mus musculus: Glycosaminoglycan metabolism	2	110	0.0051259	0.0
4810458	Mus musculus: MPS IX - Natowicz syndrome	2	110	0.0051259	0.0
4810459	Mus musculus: Mucopolysaccharidoses	2	110	0.0051259	0.0
4810460	Mus musculus: MPS IIIB - Sanfilippo syndrome B	2	110	0.0051259	0.0

Other tests

Let's have a look at the distribution of log2 fold change of genes in the leaves of the Venn diagram, as compared with that of the heart of the Venn diagram. It is expected that the leaves may have fold change different from the main DEGs.

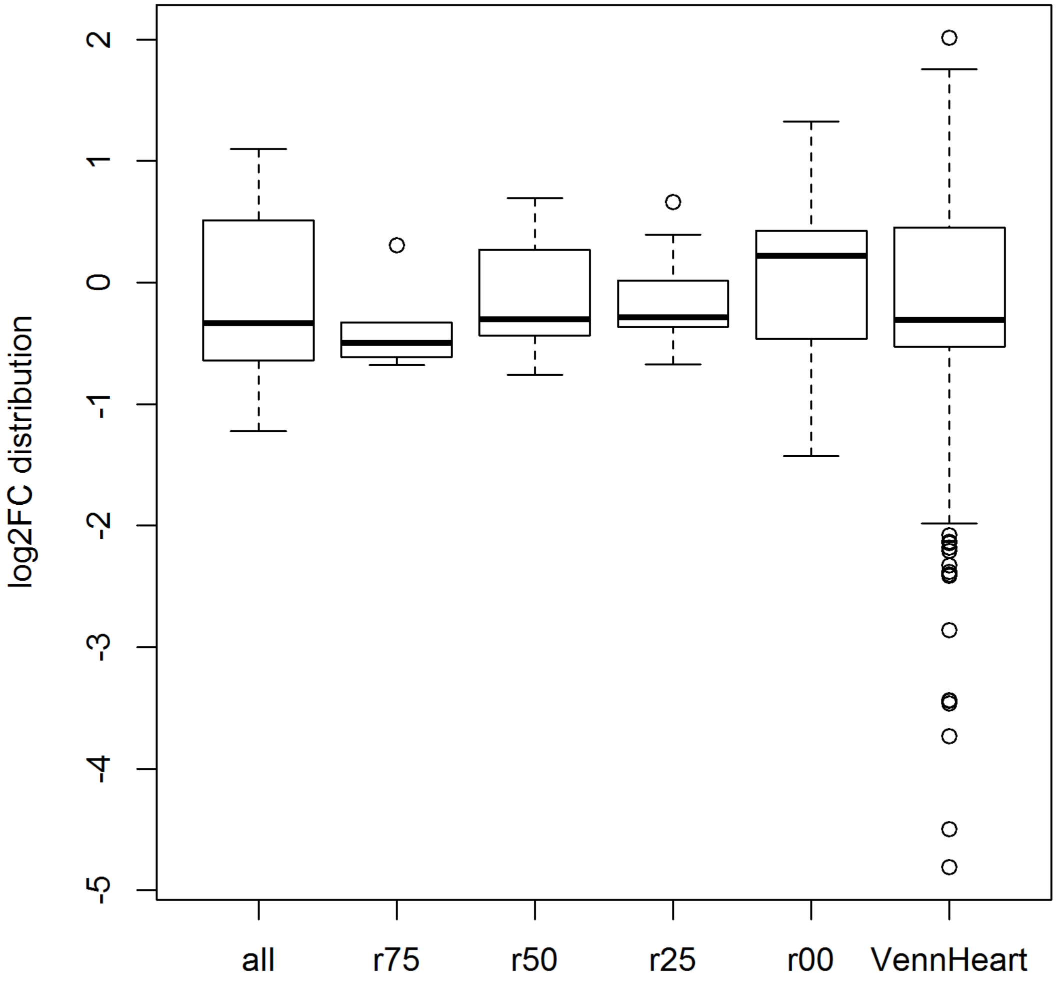

We also check the same for the expression level.

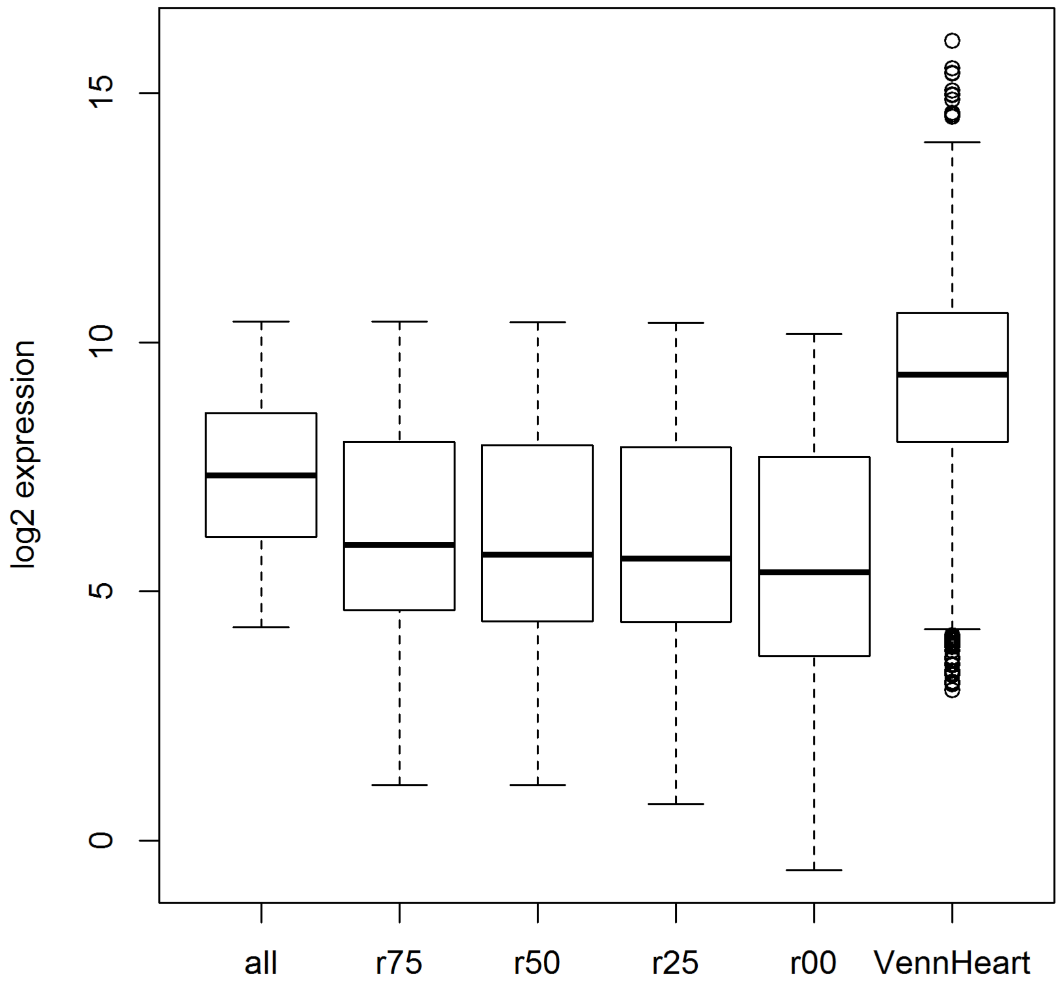

The leaves may have overall lower expression level, hence, more susceptible to the RepeatSoaker.
